# Effectiveness of interventions to prevent perinatal depression: An umbrella review of systematic reviews and meta-analysis

**DOI:** 10.1016/j.genhosppsych.2023.03.007

**Published:** 2023

**Authors:** Emma Motrico, Rena Bina, Angelos P. Kassianos, Huynh-Nhu Le, Vera Mateus, Deniz Oztekin, Maria F. Rodriguez- Muñoz, Patricia Moreno-Peral, Sonia Conejo-Cerón

**Affiliations:** a Department of Psychology, University Loyola Andalucía, Spain; b School of Social Work, Bar Ilan University, Ramat Gan, Israel; c Department of Applied Health Research, University College London, 1-19 Torrington Place, London WC1E 7HB, UK; d Department of Nursing, Cyprus University of Technology, 30 Archbishop Kyprianos, Limassol 3036, Cyprus; e Department of Psychological and Brain Sciences, George Washington University, Washington, DC, USA; f Portucalense Institute for Human Development (INPP), Department of Psychology and Education, Universidade Portucalense, Porto, Portugal; g Department of Obstetrics and Gynecology, İzmir Bakircay University, İzmir, Turkey; h Faculty of Psychology, Universidad Nacional de Educación a Distancia, (UNED), Spain; i Biomedical Research Institute of Malaga (IBIMA plataforma Bionand), Málaga, Spain; j Network for Research on Chronicity, Primary Care, and Health Promotion (RICAPPS), ISCIII, Spain; k Center for Research in Neuropsychology and Cognitive Behavioural Intervention (CINEICC), Faculty of Psychology and Educational Sciences, University of Coimbra, Portugal; l Department of Personality, Evaluation and Psychological Treatment, University of Málaga (UMA), Spain

**Keywords:** Perinatal, depression, Prevention, Randomized control trial, systematic review, meta-analysis

## Abstract

**Background:**

To date, dozens of systematic reviews (SRs) and meta-analyses (MAs) summarize the effectiveness of preventive interventions for perinatal depression. However, the results are inconclusive, making an urgent need to step up to higher levels of evidence synthesis.

**Aims:**

To summarize and compare the evidence from the SR&MA examining the effectiveness of all types of interventions for preventing perinatal depression.

**Method:**

PubMed, PsycINFO, Cochrane Database of Systematic Reviews and OpenGrey were searched from inception to December 2022. We selected SR&MA of randomized controlled trials (RCTs) that compared all types of preventive interventions for perinatal depression with control groups whose outcome was the reduction of depressive symptoms and/or incidence of new cases of perinatal depression (PROSPERO: CRD42020173125).

**Results:**

A total of 19 SRs and MAs evaluated 152 unique RCTs that included 83,408 women from 26 countries and five continents. The median effect size for any intervention was SMD = 0.29 (95% CI: 0.20 to 0.38). Exercise/physical activity-based, psychological, and any type of intervention showed median effect sizes of 0.43, 0.28 and 0.36, respectively. The degree of overlap among RCTs was slight. According to AMSTAR-2, 79% of them were rated as low or critically low-quality. The strength of evidence, according to GRADE, was poorly reported and, in most cases, was low.

**Conclusions:**

Exercise/physical activity-based and psychological interventions have a small-to-medium effect on reducing perinatal depressive symptoms. There is insufficient evidence to conclude that dietary supplements and pharmacological interventions are effective in preventing perinatal depression. There is a need for high-quality SR&MA of RCTs, mainly focusing on universal preventive interventions.

## Highlights

• This is the first umbrella review examining the effectiveness of all interventions for preventing perinatal depression
• Exercise and psychological interventions have a small-to-medium effect on reducing perinatal depressive symptoms.
• There is insufficient evidence to conclude that dietary supplements and pharmacological interventions are effective.
• There is a need for high-quality systematic reviews and meta-analyses focusing on universal preventive interventions.

## 1 Background

Depression is the most common psychiatric condition during the perinatal period. Perinatal depression is defined as a mood disorder during pregnancy and the year following birth [1,2]. Globally, it is estimated that an average prevalence of 12% of women meets the diagnostic criteria for perinatal depression disorder [3]. However, the rate has doubled due to the COVID-19 pandemic [4]. When left untreated, perinatal depression has a negative impact on the mental and physical health of women [[5], [6], [7]], their partners [8] and offspring [7,9], leaving a substantial economic burden on society. It is estimated that the average cost to society of one case of untreated perinatal depression is approximately £74,000 per mother-child pair in the United Kingdom [10] and $31,800 per mother-child pair with postpartum illness in the United States [11].

In recent years several treatment options for perinatal depression have become available, especially antidepressant medication and cognitive behavioural therapy [[13], [14], [15]]. However, not all depressed women receive appropriate treatment [1,16] and current treatments' effects are still modest [17]. Given the negative consequences of perinatal depression, effective primary preventive interventions are crucial [12,13]. Thus, preventive interventions may be considered an alternative way to reduce the burden of perinatal depression and avoid the personal suffering of women and their families.

### 1.1 Interventions to prevent perinatal depression

Preventive interventions are defined as a combination of strategies for reducing symptoms or avoiding the incidence of depression in women during the perinatal period without a clinical diagnosis [14,15]. Such interventions could target the entire population regardless of individual risk (universal prevention), a subpopulation known to be at increased risk (selective prevention), or women already showing subclinical symptoms of depression (indicated prevention) [14].

In the past two decades, hundreds of randomized control trials (RCTs) have been conducted to determine the effectiveness of preventive interventions for perinatal depression and dozens of systematic reviews and meta-analyses (SR&MA) have summarized the evidence available; however, their findings are inconclusive and conflicting. Given the rapid increase in SR&MA publishing and the contradictions among them, it has become very complex for health care practitioners and policy-makers to keep up with reviews published to adequately support clinical practice guideline development or health policy decision-making [16].

Thus, the European Network of Peripartum Depression (Riseup-PPD) [17], a multidisciplinary network of researchers dedicated to the global understanding of peripartum depression, reports that the number of clinical practice guidelines that include recommendations for preventive interventions for perinatal depression in Europe is small [18]. Therefore, there is an urgent need to step up to higher levels of evidence synthesis to determine the preventive effect of the interventions for perinatal depression.

### 1.2 An overview of the evidence for the prevention of perinatal depression is urgently needed

Umbrella reviews are the high-level synthesis of the evidence and address research questions broader in scope than those examined in individual SR&MA [19]. An umbrella review presents the most comprehensive and robust synthesis of scientific evidence to inform decision-making [20].

To the best of our knowledge, two umbrella reviews [21,22] of preventive interventions for depression in the general population have been conducted to date. One umbrella review focused exclusively on universal interventions and did not include any SR&MA focus on the perinatal period [22]. The other only included six SR&MA focused on mainly psychological interventions during the perinatal period that included randomized and nonrandomized controlled trials published before June 2020 [21]. In addition, new SR&MA on RCTs for the prevention of perinatal depression have been recently published [[23], [24], [25]]. Thus, the evidence on existing preventive interventions for depression in perinatal period remains unclear.

## 2 Aims

The present umbrella review aimed to summarize and compare the evidence from systematic reviews and meta-analyses (SR&MA) of randomized controlled trials (RCTs) examining the effectiveness of all types of interventions for preventing perinatal depression.

## 3 Method

### 3.1 Reporting and protocol registration

This umbrella review was reported according to the Preferred Reporting Items for Overviews of Systematic Reviews [26,27] and followed the Cochrane Collaboration's Guideline for overviews of reviews [19]. The protocol for this study was registered with the International Prospective Register of Systematic Reviews (PROSPERO), registration number: CRD42020173125.

### 3.2 Eligibility criteria

Studies were eligible if they met the following inclusion criteria (see Supplementary Table S1):

Participants: Women during the perinatal period (from pregnancy up to a maximum of one year postpartum) with or without the risk of developing depression were eligible. Studies focused on perinatal women with a current diagnosis of depression or being treated for a depressive episode were excluded.

Interventions: No restriction for the selection of preventive interventions was applied. Therefore, psychological (e.g., cognitive behavioural therapy), educational (e.g., self-help manual), psychosocial (e.g., social support), pharmacological (e.g., antidepressant medication), physical (e.g., exercise-based), lifestyle interventions (e.g., diet, sleep) and alternative therapies (e.g., acupuncture) among other interventions were included.

Comparators: Control groups allowed were care as usual, no treatment, waiting list, attention control or any type of placebo.

Outcomes: We selected studies whose outcomes were the prevention of perinatal depression (postpartum depression and/or prenatal depression) through the reduction of the incidence of new cases of depression and/or reduction of depressive symptomatology, assessed with validated questionnaires or standardized clinical interviews. In case any SR&MA included treatment and prevention outcomes separately, only the prevention outcomes were reported.

Design: SR&MA of RCTs were included because RCTs are the reference standard for clinical trials and provide more evidence on causality than other types of studies [28]. If the SR&MA included different types of designs than RCTs, they were excluded. SR&MA were also excluded if they only included a single RCT.

### 3.3 Information sources and search strategy

Four bibliographical databases, including PubMed, PsycINFO, Cochrane Database of Systematic Reviews and OpenGrey, were searched from inception to 2022. The initial search was conducted in April 2020, with a final search update carried out on 1 December 2022. The search strategy was first developed for PubMed using a combination of keywords and MeSH terms related to ‘perinatal period’, ‘depression’, ‘prevention’, ‘systematic review’ and ‘meta-analyses’. Then, it was adapted to the other databases. A detailed description of the search strategy is depicted in Supplementary Table S2.

The reference list of retrieved SR&MA and the list of relevant umbrella reviews were checked manually. Finally, experts in the field were consulted to identify additional relevant publications. Rayyan software [29] was used for data management.

### 3.4 Study selection

Once duplicate studies resulting from search strategies were removed, all records were reviewed for inclusion in two phases according to our eligibility criteria. First, studies were screened based on titles and abstracts, and then, the full text of the selected studies was evaluated for final inclusion. This selection process was performed by three independent reviewers (SCC, EM, VM), and disagreements were solved through discussion.

### 3.5 Data extraction process

Data were independently extracted by three pairs of reviewers (DO, EM, MFR, PMP, HNL, SCC) using a purposefully designed data extraction form. Discrepancies between the reviewers were reconciled by consensus among the team members. When necessary, reviewers contacted study authors to obtain missing data. Data extracted from each study included: author(s), year of publication, publication type (systematic review or meta-analysis), the total number of RCTs included, the total sample size in each study, characteristics of the target population, type of prevention (universal, selective, indicated), a summary description of the interventions, intervention providers, comparison, outcome(s), follow-up periods, and results on perinatal depression. Data for the included meta-analyses were also collected (pooled effect sizes with their 95% confidence intervals (CI) and heterogeneity) (reported in Table 2, Table 3).

### 3.6 Overlap of studies between systematic reviews and/or meta-analysis

Because RCTs (primary studies) are often included in more than one SR&MA, the degree of overlap within and between SR&MA was assessed using the methodology proposed by Pieper et al. [30]. The degree of overlap was calculated using the ‘corrected covered area’ (CCA). It was presented with novel graphical approaches [31]. CCA below 5% was considered a slight overlap, a CCA > 5 and ≤ 10% a moderate overlap, a CCA > 10 and ≤ 15% a high overlap, and a CCA > 15% as a very high overlap [30].

### 3.7 Data quality assessment

The methodological quality of the included SRs and MAs was evaluated using ‘A Measurement Tool to Assess Systematic Reviews’ version 2 (AMSTAR 2) [32]. AMSTAR 2 includes 16 criteria, which all indicate the quality of a SR&MA. Seven out of 16 criteria were considered as ‘critical domains’: 1) protocol registered before the commencement of the review (Item 2); 2) adequacy of the literature search (Item 4); 3) justification for excluding individual studies (Item 7); 4) risk of bias from individual studies being included in the review (Item 9); 5) appropriateness of meta-analytical methods (Item 11); 6) consideration of the risk of bias when interpreting the results of the review (Item 13), and 7) assessment of the presence and likely impact of publication bias (Item 15). Therefore, we classified SR&MA with no or one noncritical weakness as ‘high’ confidence in the results; studies with more than one noncritical weakness as ‘moderate’; studies with one critical flaw with or without noncritical weaknesses as ‘low’; and studies with more than one critical flaw with or without noncritical weaknesses as ‘critically low’ [32]. Three pairs of reviewers evaluated the quality assessment independently (VM, EM, MFR, PMP, AK, SCC), and any discrepancies were solved through discussion until a consensus was reached.

### 3.8 Quality of the evidence

The strength of evidence for each meta-analytic effect was reported based on the Gradings of Recommendations, Assessment, Development and Evaluation system (GRADE) [33]. Assessment of the quality of the evidence considers five aspects: risk of bias, publication bias, imprecision (random error), inconsistency and indirectness [34]. Specifically, we evaluated the strength of evidence of each meta-analysis based on the corresponding results from the SR&MA.

### 3.9 Data synthesis

A meta-level narrative synthesis was performed for all included SRs and MsA. For studies performing a meta-analysis, we re-analyzed and converted the pooled effect sizes and their corresponding 95% CI into standardized mean differences (SMDs) using a random effects model. Continuous effect sizes, such as Hedges' g and categorical effect sizes, such as risk ratio (RR), were presented in the form of SMD. We constructed a forest plot with the SMDs and their corresponding 95% CI in perinatal depression. Positive SMDs indicated a better outcome in the intervention group. The overall meta-analytical effect side was interpreted following Cohen's proposal [35]. Cohen classified effect sizes as small (0.2), medium (0.5) and large (0.8) (Cohen, 1989). All analyses were undertaken using the Comprehensive Meta-Analysis (CMA) software package (V.2.2.021).

## 4 Results

### 4.1 Search results

**Fig. 1:**
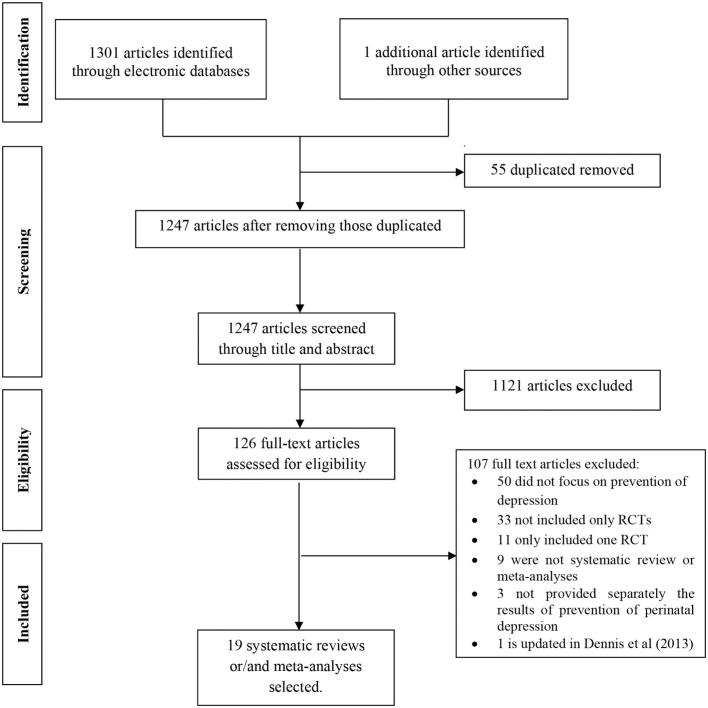
Flow-chart of excluded and included systematic reviews and meta-analyses.

A total of 1247 articles were identified after excluding duplicates. Of these, 126 articles were included for full-text review and 19 different SRs and MAs met the inclusion criteria [[23], [24], [25],[36], [37], [38], [39], [40], [41], [42], [43], [44], [45], [46], [47], [48], [49], [50], [51]] (see Fig. 1). The references of the 107 studies excluded with reasons are available in Supplementary Table S3.

### 4.2 Characteristics of the included studies

The characteristics of the 19 included SRs and MAs are presented in Table 1. Of them, 6 were SR and 13 also performed MA (62.5%). All SRs and MAs were published between 2005 and 2022. The SR&MA included 152 (62.1%) unique RCTs (from a total of 201 RCTs included) and 83,408 women in the perinatal period. The RCTs included in the SR&MA were carried out in 26 countries on five continents. Most of the RCTs were conducted in USA (*N* = 51), Australia and New Zealand (*N* = 25), China (*N* = 23) and the United Kingdom (*N* = 18). The list of the countries is available in Supplementary Table S4. Only two SR&MA [23,36] provided information regarding ethnic minorities. One SR&MA [23] included five RCTs focused on African American women, American Indians, and Latinas in the United States. In addition, one SR [36] included five RCTs focusing on the American Indian population, eight on the African American population, and four on the Hispanic population.

**Table 1:** Details of the systematic reviews and meta-analysis included (*N* = 19).

Author – year	Type of Study	Last date search	Total sample (included trials)	Target population	Type of prevention	Intervention	Intervention provider	Comparison	Outcome measures	Follow-up (N: follow-up months)
Boath et al. 2005 a	Systematic review	June 2003	8310 (20 trials)	Pregnant and postpartum women	Universal Indicated Selective	Psychological (IPT), psychoeducational, and psychosocial (social support, home visiting), antidepressants, and hormonal	Not reported	Usual care, placebo.	Postpartum depressive symptoms and incidence of postpartum depression	3: < 3 months 3: 4–6 months 15: < 6 months
Carter et al. 2019 b	Meta-analysis	June 2017	1271 (7 trials)	Postpartum women (4–52 weeks)	Universal	Exercise and physical activity-based interventions	Qualified and nonqualified service providers	No intervention, active control, usual care	Postpartum depressive symptoms	6: < 3 months 1: 4–6 months
Cluxton-Keller & Bruce, 2018^c^	Meta-analysis	November 2017	919 (5 trials)	Pregnant and postpartum women (6 months) and partners.	Universal Indicated	Family therapy (IPT, humanistic, CBT, psychoeducation) and systemic intervention.	Mental health professionals (social workers, doctoral-trained licensed marriage and family therapists, psychologists, psychiatrists, psychology trainees, and trained maternal and child health nurses)	Standard care, treatment as usual, wait-listed, or not care conditions	Perinatal depressive symptoms	Not reported.
Dennis & Dowswell, 2013	Meta-analysis	December 2012	51,356 (30 trials)	Pregnant and postpartum women (< 6 weeks)	Universal Selective Indicated	Psychological (debriefing, IPT and CBT) and psychosocial (home visits, educational programs, social support)	Physicians, nurses, midwives, other health care providers	No intervention, active control, usual care	Postpartum depressive symptoms	13: < 3 months 10: 4–6 months
Goodman et al. 2018^d^	Meta-analysis	Not provided	24,690 (14 trials)	Pregnant women	Universal Selective Indicated	Psychological (CBT, behavioural parent training, couple-focused, psychosomatic) psychoeducational, psychosocial (social support) yoga/massage, nutrition/mineral supplementation	Not reported	Other interventions, active control, usual care	Perinatal depressive symptoms	Not reported
Lin et al. 2018^e^	Meta-analysis	September 2017	207 (2 trials)	Pregnant and postpartum women (23 < 52 weeks)	Selective Indicated	Self-help intervention: stress management and exercise-based	Not proceed	Face-to-face, usual care	Postpartum depressive symptoms	2: < 3 months
Martin-Gomez et al. 2022	Meta-analysis	August 2020	4958 (17 trials)	Pregnant and postpartum women (< 52 weeks)	Universal Selective Indicated	Psychological, including those with psychoeducational and psychosocial approaches.	Health professionals (nurses, therapists, midwives, gynecologists, psychiatrists, clinical psychologists and clinical social workers), predoctoral and postdoctoral students in clinical psychology, paraprofessionals from the same cultural background as the participants, social workers and other trained interventionists.	Usual care, attention control, waiting list or no intervention	Postpartum depressive symptoms and incidence of postpartum depression	9: 4–6 months 8: < 6 months
McCurdy et al. 2017^f^	Meta-analysis	June 2016	1327 (6 trials)	Postpartum women	Universal	Exercise-based intervention (Supervised or unsupervised)	Not reported	Control group inactive or a waitlist group	Postpartum depressive symptoms	Not reported
Miller et al., 2013	Systematic review	April 2013	305 (2 trials)	Pregnant and postpartum women	Universal Indicated	Omega-3 supplementation	Not reported	Placebo	Postpartum depressive symptoms and incidence of postpartum depression	2: < 3 months
Missler et al. 2021^g^	Meta-analysis	November 2018	2369 (10 trials)	Pregnant women	Universal	Psychological (CBT and IPT) and psychoeducational	Mental health care professionals (psychologists), obstetricians and midwives.	Usual care, placebo, waiting list.	Postpartum depressive symptoms	Not reported
Molyneaux et al., 2018	Systematic review	February 2018	81 (2 trials)	Pregnant and postpartum women (< 6 weeks)	Selective	Antidepressant medication (nortriptyline and sertraline)	Not reported	Placebo	Postpartum depressive symptoms and incidence of postpartum depression	2: 4–6 months
Sangsawang et al., 2018	Systematic review	March 2017	2660 (13 trials)	Adolescent pregnant and postpartum women	Selective Indicated	Psychological (psycho-educational and CBT, group IPT) and psychosocial (home visits, prenatal/postnatal educational programs, social support interventions, early intervention programs, infant massage training)	Public health nurse, trained home visitor, pamphlet, videotape, para-professional, trained provider from the local community and trained prenatal care provider	Usual car, home visits for breastfeeding lessons, home visits for breastfeeding and nutrition education programs, education support	Postpartum depressive symptoms and incidence of postpartum depression	2: Not reported 2: < 3 months 3: 4–6 months 6: < 12 months
Sasaki et al. 2020	Systematic review	January 2019	836 (2 trials)	Pregnant women	Universal	Psychoeducational intervention focused on maternal or infant sleep	Not reported	No treatment, waitlist control, treatment as a usual or active control.	Postpartum depressive symptoms	1: < 3 months 1: 4–6 months
Shaw et al., 2006^h^	Systematic review	2005	7997 (10 trials)	Postpartum women (< 52 weeks)	Universal Selective	Psychosocial (nurse home visits, conference care, telephone support, standardized debriefing session, self-help manual and support group invitation)	Public health nurses, mothers who had previously experienced postpartum depression, trained midwives, health visitors, social workers, nurse practitioner	Usual care, usual information, brochure	Postpartum depressive symptoms	7: < 3 months 4: 4–6 months
Suradom et al., 2021^i^^j^	Meta-Analysis	May 2020	3205 (9 trials)	Pregnant and postpartum women	Universal Selective Indicated	Intervention–n-3 PUFA supplementation.	Not provided	Placebo	Postpartum depressive symptom	6: < 3 months 3: 4–6 months
Yasuma et al. 2020	Meta-analysis	January 2019	7416 (18 trials)	Pregnant women	Universal	Psychological (CBT-based approach, mindfulness, IPT and psychoeducation)	Medical professionals or psychologists.	Usual care	Antenatal and postpartum depressive symptoms and incidence of depression	16: < 3 months 2: 4–6 months
Yin et al., 2020^k^	Meta-analysis	February 2019	2304 (14 trials)	Pregnant and postpartum women (< 52 weeks)	Universal Selective Indicated	Psychological (CBT-based, mindfulness, IPT and psychoeducation), psychosocial, exercise-based, and traditional Chinese medicine	Obstetricians, psychiatrists, nurses, midwives, psychologist	Usual care	Antenatal and postpartum depressive symptoms and incidence of depression	12: < 3 months 2: 4–6 months
Zhou et al., 2020^k^	Meta-analysis	December 2019	820 (5 trials)	Postpartum women	Universal Selective	mHealth (psychological, psychoeducational, and exercise-based)	Midwives, nurses	Usual patient care and the final EPDS assessment time ranged from 8 to 24 weeks.	Postpartum depressive symptoms	2: < 3 months 3: 4–6 months
Zhu et al., 2021^l^	Meta-analysis	April 2021	1445 (14 trials)	Pregnant women	Universal	Yoga, massage, music, and exercise	Certified prenatal yoga instructors and therapists.	Usual care	Antenatal depressive symptoms	Not reported

**Note:** CBT: Cognitive Behavioural Therapy; IPT: Interpersonal Therapy.

a This systematic review included one nonpublished study.

b Only preventive interventions for depression are included. Total sample: 1428 participants (17 trials).

c Only preventive interventions for depression are included. Total sample: 1026 participants (7 trials).

d Only preventive interventions for depression are included. Total sample: 27342 participants (25 trials). Prevention studies included 14 RTCs and 16 comparisons.

e Only preventive interventions for depression are included. Total sample: 1038 participants (9 trials).

f Only preventive interventions for depression are included. Total sample: 1327 (16 trials).

g Only preventive interventions for depression are included. Total sample: 2559 (12 trials).

h Only preventive interventions for depression are included. Total sample: 14436 (22 trials).

i Only preventive interventions for depression are included. Total sample: 3181 (12 trials).

j Only preventive interventions for depression are included. Total sample: 4673 (26 trials).

k Only preventive interventions for depression are included. Total sample: 2424 (11 trials).

l Only preventive interventions for depression are included. Total sample: 2841 (24 trials*).*

Regarding the target population, five SRs and MAs were focused on pregnant women [24,25,37,40,49], four on postpartum women [38,42,46,51] and ten on both pregnant and postpartum women [23,36,39,41,[43], [44], [45],^47^,^48^,^50^]. All except one were focused on adult women, and one was conducted only with adolescents [36]. Most of the SRs and MAs (k = 12; 63,15%) covered at least two types of prevention approaches (universal, selective or indicated) [23,36,38,39,41,42,45,[47], [48], [49], [50], [51]], six SRs and MAs focused exclusively on universal prevention [24,25,37,40,46,51] and one on selective prevention [43].

Regarding the type of the preventive intervention that was carried out, eight SRs and MAs reviewed psychological interventions, including those with a psychoeducational and psychosocial approaches [23,24,37,38,40,41,47,48]. Two analyzed exercise-based interventions [46,51], two dietary supplements [39,44], one pharmacological intervention [43], and five included any type of intervention [25,42,45,49,50]. Regarding comparator groups, twelve SRs and MAs included different types of groups, such as usual care, no intervention or waiting list [23,[36], [37], [38],[45], [46], [47], [48], [49], [50], [51], [52]], four included only usual care [25,[40], [41], [42]] and three included placebo [39,43,44]. Most interventions were provided by health professionals (nurses, therapists, midwives, gynecologists, psychiatrists, clinical psychologists, and clinical social workers), nonprofessional service providers and other trained interventionists.

Approaching the outcome on perinatal depression, 14 SRs and MAs evaluated the reduction of depressive symptoms in the postpartum period [23,24,36,[38], [39], [40],[42], [43], [44], [45], [46],^48^,^50^,^51^,^53^,^54^]. Only five SRs and MAs assessed depressive symptoms during the antenatal period [25,37,41] or the whole perinatal period [47,49]. Furthermore, seven RSs and MAs reported the incidence of postpartum depression [23,36,40,41,[43], [44], [45]]. The last follow-up evaluation was carried out from 48 h after delivery (interventions during pregnancy) to 12 months postpartum. No review included follow-ups lasting >12 months.

### 4.3 Overlap of studies between systematic reviews and/or meta-analyses

**Fig. 2:**
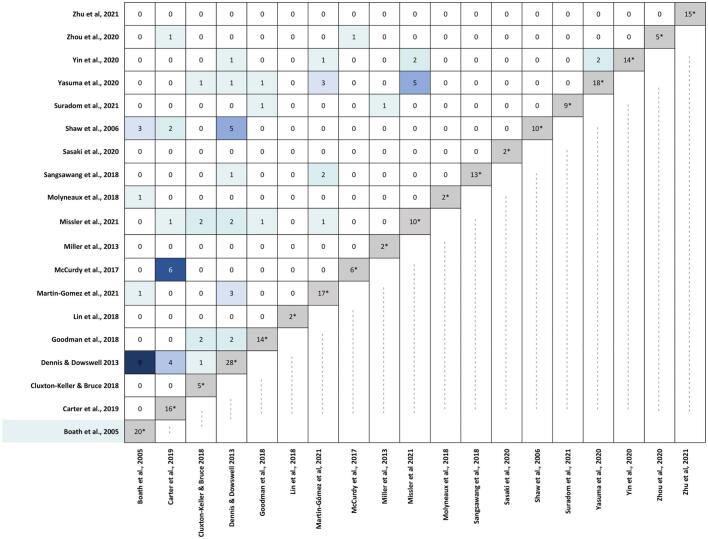
Overlap between the systematic review and/or meta-analysis included in the umbrella review. *Note*. *: The total number of primary studies (RCTs) included in the systematic review and/or meta-analysis. White = 0 RCTs duplicate; Very light green: 1 RCT duplicate; Light green: 2 RCTs duplicate; Very light blue: 3 RCTs duplicate; Light blue: 4 RCTs duplicate; Blue: 5 RCTs duplicate; Dark blue: 6 RCTs duplicate; Very dark blue: 9 RCTs duplicate. (For interpretation of the references to colour in this figure legend, the reader is referred to the web version of this article).

This study included a total of 152 (62.1%) unique RCTs. Of these, 113 RCTs were cited in one SR&MA, 31 were included in two, and 7 were included in three and one in five SR&MA. The total CCA was 2%, indicating a slight degree of overlap. See details of the overlap between SR&MA in Fig. 2.

### 4.4 Methodological quality

The methodological quality of the included SRs and MAs is described in Table 2. Most of the SRs and MAs (79%) were classified as low or critically low-quality using AMSTAR-2, mainly due to the nonexistence of a list of excluded studies with associated reasons and to the fact that authors did not discuss the impact of risk of bias when interpreting the results. However, four SRs and MAs presented high quality [23,43,44,48].

**Table 2:** Methodological quality of the included systematic reviews and meta-analyses (N = 19).

Author (Year)	AMSTAR-2																Qualitative appraisal
	Item 1	**Item 2**	Item 3	**Item 4**	Item 5	Item 6	**Item 7**	Item 8	Item 9	Item 10	**Item 11**	Item 12	**Item 13**	Item 14	**Item 15**	Item 16	
Boath et al., 2005	−	**−**	−	**+/−**	+	+	**+**	+/−	−	−	**NM**	NM	**−**	+	**NM**	−	CL
Carter et al., 2019	+	**+**	+	**+**	+	+	**-**	+	+	−	**+**	+	**+**	+	**+**	+	L
Cluxton-Keller & Bruce, 2018	+	**+**	−	**+/−**	+	−	**−**	+	+	−	**+**	−	**−**	+	**−**	+	CL
Dennis & Dowswell, 2013	+	**+**	+	**+**	+	+	**+**	+	+	−	**+**	+	**+**	+	**+**	+	H
Goodman et al., 2018	+	**+**	+	**−**	+	−	**−**	−	+	−	**+**	+	**+**	+	**+**	−	CL
Lin et al., 2018	+	**−**	+	**+/−**	+	+	**−**	+	−	−	**+**	+	**+**	+	**+**	+	CL
Martin- Gomez et al. 2022	+	**+**	+	**+**	+	+	**−**	+	+	−	**+**	+	**+**	+	**+**	+	H
McCurdy et al., 2017	+	**−**	+	**+/−**	+	+	**−**	+/−	+/−	−	**+**	+	**+**	+	**+**	+	CL
Miller et al., 2013	+	**+**	+	**+**	+	+	**+**	+	+	−	**NM**	NM	**+**	+	**NM**	+	H
Missler et al. 2021	+	**+**	−	**+/−**	+	+	**−**	−	+	−	**+**	+	**+**	+	**+**	+	L
Molyneaux et al., 2018	+	**+**	+	**+**	+	+	**+**	+	+	+	**NM**	NM	**−**	+	**NM**	+	H
Sangsawang et al., 2018	−	**−**	−	**+/−**	+	+	**−**	+	+	−	**NM**	NM	**+**	−	**NM**	+	CL
Sasaki et al. 2020	+	**+/−**	−	**+**	+	+	**−**	+	+	−	**NM**	NM	**+**	+	**NM**	+	L
Shaw et al., 2006	−	**+/−**	−	**+/−**	−	−	**−**	+/−	+/−	+	**NM**	NM	**−**	−	**NM**	−	CL
Suradom et al., 2021	+	**+/−**	−	**+**	+	+	**−**	+	+	−	**+**	+	**+**	+	**+**	+	L
Yasuma et al., 2020	+	**+**	−	**+/−**	+	+	**−**	+	+	−	**+**	+	**−**	−	**+**	+	L
Yin et al., 2020	+	**+**	−	**+/−**	+	+	**−**	+	+	−	**+**	+	**+**	+	**+**	+	L
Zhou et al. 2020	+	**−**	−	**+/−**	+	+	**−**	+	+	−	**+**	−	**−**	+	**−**	+	CL
Zhu et al. 2021	+	**−**	+	**+/−**	+	+	**−**	−	−	−	**+**	−	**−**	−	**−**	+	CL

NM = No meta-analysis conducted; + = Yes; − = No; +/− = Partial Yes; H = High confidence; M = Moderate confidence; L = Low confidence; CL = Critically low confidence.

### 4.5 Quality of the evidence

The strength of evidence, according to the GRADE system [33], was measured in five of the 17 SRs and MAs included [23,25,42,43,46]. The grading of the quality of evidence was very low [25,43], low [46], moderate [23] and high [42].

### 4.6 Effectiveness of the interventions to prevent perinatal depression

#### 4.6.1 Overall effect

**Fig. 3:**
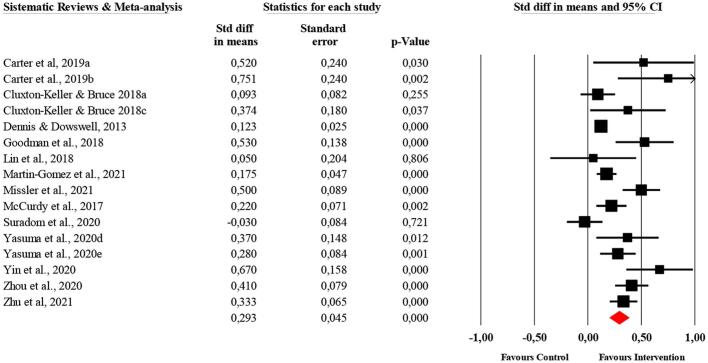
Forest-plot of SR&MA of all types of preventive interventions for perinatal depression. *Note*. Weights are from random effects analysis. (a) Universal interventions; (b) targeted interventions; (c) Indicated interventions; (d) antenatal depression; (e) postnatal depression.

Fig. 3 shows the results of the included meta-analyses. Thirteen meta-analyses reported SMDs ranging from 0,75 [46] to −0,03 (Suradom et al., 2021). The pooled SMD was 0.29 (95% CI: 0.20 to 0.38; p 〈0,000) for the random model, indicating that interventions to prevent depression in the perinatal period had a small and statistically significant effect on the reduction in perinatal depressive symptomatology.

Of the six SRs that provided narrative results, two of them suggested contradictory results, as almost half of the included RCTs showed that the interventions to prevent perinatal depression were effective and the other half did not [36,45]. The four remaining SRs concluded that there was insufficient evidence in favour of the preventive intervention [37,38,43,44].

#### 4.6.2 Psychological interventions

Six MAs and two SRs that included 81 unique RCTs [23,24,37,38,40,41,47,48] were focused on psychological interventions.

The pooled SMD for psychological preventive interventions was 0.28 (95% CI: 0.16 to 0.39). The largest effect size was SMD = 0.67 [41]. Five out six interventions had a significant effect size [23,24,40,48,56]. However, one meta-analysis reported effective results in the single RCT for indicated prevention (SMD = 0.37), but not for the pooled results of four universal prevention RCTs [47].

The two SRs showed contradictory and inconclusive findings [37,38]. One SR that included two RCTs found that behavioural knowledge and skills for optimizing sleep hygiene during pregnancy had a statistically significant reduction in postpartum depressive symptoms in one RCT but no significant differences were found in the other [37,38]. Furthermore, the other SR suggested inconclusive findings, as only 46% of the RCTs were statistically significant in reducing depressive symptoms [38]. See Table 3 and Fig. 3.

**Table 3:** Main findings of the systematic reviews and meta-analysis that were included in the umbrella review (N = 19).

Authors (year of publication)	Main findings	Effect estimates (if a meta-analysis was performed)	GRADE system
**Psychological**			
Cluxton-Keller & Bruce, 2018^a^	Four universal preventive RCTs and one indicated preventive RCT were included in the meta-analysis. The indicated preventive family therapeutic intervention was effective in reducing postpartum depressive symptoms, while universal preventive interventions did not find statistically significant reductions in perinatal depressive symptoms. The quality ratings for the included RCTs ranged from moderate to high.	SMD (g) = −0.37, 95% CI = [−0.72, −0.02]; z = 2.07, *p* = 0.04 (indicated preventive interventions) SMD (g) = −0.09, 95% CI = [−0.25, 0.06]; z = 1.13, *p* = 0.26 (Universal preventive interventions)	Not assessed
Dennis & Dowswell, 2013	Twenty-eight studies showed that women receiving psychosocial or psychological intervention were significantly less likely to develop postpartum depression than those receiving standard care. The methodological quality of the included trials was good to excellent.	RR = −0.78, 95% CI = [0.66 to 0.93], *p* ≤ 0.05, SMD = 0.12	Not assessed
Martin-Gómez et al., 2022	The results from this SR were derived from 17 RCTs, 15 of them were included in the meta-analysis. Psychological interventions had a very small effect on preventing PPD in nondepressed women, and this result was robust in the sensitivity analyses. Meta-regression found that interventions focused on primiparous women were more effective than those focused on primiparous and multiparous women.	SMD (g) = −0.17, 95% CI = [−0.26 to −0.08], p < 0.001	Moderate
Missler et al., 2021^a^	The results of 10 RCTs showed a moderate effect of psychological interventions for the universal prevention of depressive symptoms. The quality ratings for the included RCTs ranged from low to high.	SMD (d) = 0.50, 95% CI = [0.32 to 0.67]	Not assessed
Sasaki et al., 2020	The effectiveness of psycho-educational interventions was supported by one out of two RCTs. This study found limited evidence to support the effectiveness of psycho-educational interventions focused on maternal or infant sleep for pregnant women to prevent the onset of antenatal and postnatal depression.	Not performed	Not assessed
Shaw et al., 2006^a^	Postpartum psychosocial interventions were supported by 3 out of 10 RCTs. These 3 RCTs showed a decrease in depressive symptoms in psychosocial groups compared to control groups. There is insufficient evidence to conclude that postnatal women may benefit from postpartum psychosocial interventions.	Not performed	Not assessed
Yasuma et al., 2020	A total of eleven studies were included in the meta-analysis. There was a significant effect of antenatal psychological intervention on antenatal and postnatal depression with moderate to high heterogeneity. The methodological quality of the studies was low.	SMD (g) = 0.28, 95% CI = [0.11 to 0.44], *p* = 0.01 (antenatal depressive symptoms) SMD (g) = 0.37, 95% CI = [0.08 to 0.66], *p* < 0.001 (postpartum depressive symptoms)	Not assessed
Yin et al., 2020^a^	Psychosocial preventive interventions in China were evaluated in 14 RCTs to reduce perinatal depressive symptoms. The subgroup meta-analysis showed a statistically significant reduction of perinatal depressive symptoms in the combined results. The methodological quality of the included studies was low to moderate.	SMD (g) = −0.67 CI = [−0.98 to −0.36], p < 0.001	Not assessed

**Physical activity**			
Carter et al., 2019^a^	Both universal (assessed in seven RCTs) and targeted exercise-based interventions (evaluated in 9 RCTs and ten comparisons) was effective in reducing depressive symptoms in postpartum women. Targeted preventive interventions yielded a greater effect side compared to universal preventive interventions. The RCTs included participants with mild to moderate symptoms of depression at baseline. The methodological quality of the included studies was low to moderate.	SMD (g) = −0.75, 95% CI = [−1.22 to −0.28], *p* = 0.002 (Targeted preventive interventions) SMD (g) = −0.52, 95% CI = [−0.99 to −0.05], *p* = 0.03 (Universal preventive interventions)	Low
McCurdy et al., 2017	Preventive exercise-based interventions in the postpartum period were assessed in six RCTs showing a small preventive effect in the reduction of postpartum depressive symptoms. The methodological quality of the RCTs presented some limitations.	SMD (g) = −0.22, 95% CI = [−0,36 to −0,08], p = 0.002	Not assessed

**Dietary supplementation**			
Miller et al., 2013	One of the two studies showed a favourable effect of selenium on mean EPDS scores compared with a placebo, but this effect did not reach statistical significance. The other one showed no significant effect of docosahexaenoic acid (DHA) and eicosapentaenoic acid (EPA) on depressive symptoms or rates of diagnosis of major depressive disorders compared with the placebo. One RCT presented a high methodological risk of bias, and the other had a low risk of bias.	Not performed	Not assessed
Suradom et al., 2020^a^	Omega-3 polyunsaturated fatty acid (n-3 PUFA) supplementation did not show a statistically significant superiority over placebo in reducing postpartum depressive symptoms in women. Overall, the included RCTs presented a low risk of bias.	SMD (g) = −0.03, 95% CI = [−0.20 to 0.13], *p* > 0.05	Not assessed

**Pharmacological**			
Molyneaux et al., 2018	Two RCTs examined the effect of antidepressants versus placebo for the prevention of postpartum depression. The results showed that nortriptyline was not more effective than placebo in preventing postpartum major depressive disorder (RR = 0.96) in one RCT. The second RCT compared sertraline with a placebo and found some evidence that sertraline may be effective in preventing postpartum depression, but the sample size was extremely small, and the risk ratio was just below statistical significance (RR = 0.14). The risk of bias was low or unclear in most domains for both RCTs.	Not performed	Very low

**Mixed interventions**			
Boath et al., 2005^a^	Nine (seven psychological and supportive interventions, one antidepressant RCT and one calcium carbonate intervention trial) out of 21 interventions (included in 20 RCTs published) demonstrated short-term success (less than four months follow-up). Nevertheless, none provided evidence of long-term success in reducing postpartum symptoms. The risk of bias in the RCTs was not assessed.	Not performed	Not assessed
Goodman et al., 2018^a^	A meta-analysis of 14 RCTs explored a wide range of preventive interventions, including psychological, psychosocial, yoga, massage, and mineral supplements. The results showed that interventions to prevent depression during pregnancy were associated with reducing depressive symptoms, with a medium effect size. Most RCTs were of high quality (low risk of bias).	SMD (g) = 0.53, 95% CI [0.26 to 0.80], p = 0.38^b^	Not assessed
Lin et al., 2018^a^	Two self-help intervention studies (stress management and exercise-based) were included. The MA showed that self-help preventive interventions did not differ significantly from control conditions in reducing postpartum depressive symptoms. The methodological quality was high in two RCTs and moderated in the remaining one.	SMD (g) = −0.29, 95% CI [0.70 to 0.12], *p* = 0.151	Not assessed
Sangsawang et al., 2018	Six of 13 studies were found to significantly prevent PPD in adolescent mothers. These six effective preventive programs (two based on psychoeducational, three on psychosocial and one on infant massage interventions) successfully prevented PPD among adolescent mothers by having lower depression scores or incidence of PPD compared to the controlled conditions. Most of the RCTs were classified as good quality.	Not performed	Not assessed
Zhou et al., 2020^a^	The meta-analysis showed that mHealth interventions had a significant effect on the reduction of postpartum depression symptoms compared with control groups. The risk of bias was low in all the RCTs.	MD = −1.02, 95% CI [−1.40 to −0.64], *p* < 0.0001	High
Zhu et al., 2021^a^	A total of 14 trials (15 comparisons) of yoga, massage, music, and exercise interventions for the prevention of antenatal depression were included. Regarding yoga, two studies provided evidence, and two provided no evidence for reducing depression. The only study on massage did not show results in favour of the intervention. All exercise interventions were associated with a reduction of antenatal depression symptoms. MA of music interventions showed a significant large effect in favour of music in preventing antenatal depression.	SMD (g) = −1.35, 95% CI [−1.87, −0.84], p < 0.0001 (Music intervention)	Low (yoga, music, exercise) and very low (music)

**Note.** GRADE: Grading of Recommendations Assessment, Development and Evaluation tool; SMD: standardized mean difference; RCT: randomized controlled trial; MD = median deviation; SMD: standardized median deviation; g: Hedges' g; d: Cohen's d.

a Only preventive interventions for depression are included.

b The result of the effect size corresponds to subgroup analysis.

The overlap of RCTs among these eight SRs and MAs was slight (CCA = 4%). The quality of evidence according to the GRADE system was reported in only one MA and was judged as moderate [23].

#### 4.6.3 Exercise/physical activity based

Two MAs that included 15 unique RCTs were focused on different types of exercise-based interventions such as aerobic exercise, resistance training, flow yoga and stretching [46,51]. The pooled SMD was 0.43 (95% CI: 0.09 to 0.77). The largest effect size was SMD = 0.75 [46]. Both interventions had a significant effect size [46,51]. See Table 3 and Fig. 3.

The overlap of RCTs among these two MAs was very high (CCA = 62.5%). Five of thirteen RCTs were included in both MAs. The quality of the evidence following the GRADE system was low in Carter et al. [46] and was not reported in the other study [51].

#### 4.6.4 Dietary supplementation

One MA and one SR that included 10 unique RCTs [39,44] that examined dietary supplementation interventions. These interventions included selenium, docosahexaenoic acid (DHA), eicosatetraenoic acid (EPA) and omega-3 polyunsaturated fatty acid (n-3 PUFA).

The unique MA reported a no significant effect on dietary supplementation interventions on the reduction of postpartum depressive symptoms compared with placebo (SMD = -0.03; 95% CI: −0.19 to 0.13) [39]. Neither of the two studies included in the Cochrane SR that compared DHA and EPA with placebo [44]. See Table 3 and Fig. 3.

The overlap of RCTs was moderate (CCA = 10%). One out 11 RCTs was included in both the SR&MA. The quality of the evidence was not measured in any SR&MA of this type of intervention.

#### 4.6.5 Pharmacological interventions

One SR that included two RCTs examined the effect of antidepressants versus placebo for the prevention of postpartum depression [43]. The results showed no significant effectiveness of antidepressants in preventing postpartum depression. The certainty of the evidence was considered very low based on the GRADE system [43]. See Table 3.

#### 4.6.6 Any type of intervention

Four MAs [25,42,49,57] and two SRs [36,45] with a total of 69 unique RCTs assessed any type of intervention in their reviews. All of them included psychological and/or exercise-based interventions, but some also included dietary supplementation, pharmacological interventions, music therapy and massage.

The pooled SMD for any preventive interventions was 0.36 (95% CI: 0.24 to 0.48). The largest effect size was SMD = 0.53 [49]. Three of the four MAs had a significant effect size [25,42,49]. The SRs by Boath et al. [45] and Sangsawang et al. [36] reported respectively that 45% (20 RCTs) and 46% (6 RCTs) of the total of trials included, respectively, found a statistically significant results to prevent postpartum depression. See Table 3 and Fig. 3.

There was no overlap between the RCTs in these six SR&MA (CCA = 0%). According to GRADE, the quality of the evidence was high in the MA by Zhou et al. [42] and very low and low in the SR&MA by Zhu [25]. The quality of the evidence was not reported in the remaining SR&MA.

## 5 Discussion

### 5.1 Summary of findings

To the best of our knowledge, this is the first umbrella review to provide a comprehensive overview of all types of preventive interventions for perinatal depression. This umbrella review found that the meta-analytic evidence base for the prevention of perinatal depression supports exercise/physical activity based (SMD = 0.43) and psychological interventions (SMD = 0.28). We also found that most SR&MA of any types of interventions were also effective (SMD = 0.36), but all included mainly exercise/physical activity-based and psychological interventions plus some other interventions (e.g., massage) and the effectiveness of the interventions was not compared with each other. Insufficient evidence was found to conclude that dietary supplements and pharmacological interventions were effective in preventing perinatal depression.

These findings were derived from 19 SRs and MAs (13 MAs and 6 SRs) including 152 unique RCTs (from a total of 201 RCTs included) from 26 countries on five continents. We found slight overlap between all RCTs included. According to AMSTAR-2, most of the SR&MA (79%) were classified as low or critically low-quality. Finally, the strength of evidence, according to GRADE, was reported in five SRs and MAs, being three very low and low.

### 5.2 Strengths

Our strict inclusion criteria, analyzing only reviews that included exclusively RCTs with the objective of preventing perinatal depression, helps to ensure that the results are based on the best quality of evidence available. Our study included a large number of SRs and MAs and incorporated a large number of RCTs that included a wide range of preventive interventions from different settings and countries. Therefore, our results are quite generalizable and support its external validity. >73.6% of the SRs and MAs included in our review have been conducted in the past five years, and three were conducted in the last year (2021−2022), therefore our results are based on relatively current SRs and MAs.

We used multiple complementary electronic databases (including OpenGrey), expert consultation and supplementary hand searching. The variety of databases applied, combined with the broad range of search terms and wide scope of study publication language, contributed to a highly sensitive search. We applied rigorous methodology (e.g., PRIOR) to the systematic review process and the evaluation of the methodological quality of the reviews (e.g., AMSTAR-2) and the study was prospectively registered in PROSPERO.

### 5.3 Limitations

Several limitations should be considered when interpreting the results. First, some RCTs were included more than once in the selected SR&MA, which could have skewed some results. However, we found that the overall overlap of RCTs among SR&MA was slight, CCA of 2% [30]. The highest degree of overlap was reported in the two MA on exercise-based interventions [46,51], which could have led to the overrepresentation of the included study results. However, this should not preclude the presentation of overall trends of results.

Second, most of the RCTs included in the SR&MA were conducted in high-income countries. Available information regarding the inclusion of racial/ethnic minorities is scarce and limited in high-risk groups, especially racial/ethnic minority populations in the USA. Therefore, the findings of this general review may not be generalizable to diverse populations across different cultures and countries, particularly in low- and middle-income countries.

Third, another limitation relates to the overall low methodological quality of the SR&MA included, based on AMSTAR-2 ratings. Furthermore, the overall quality of the evidence following GRADE was only reported in few (26%) of the studies. Thus, more high-quality SR&MA studies are needed.

Fourth, only two SRs and MAs [23,38] excluded RCTs with depressed participants at baseline. None of the remaining SR&MA excluded women with depression at the beginning of the study. Consequently, the results do not allow a clear distinction to be made between prevention effectiveness and treatment effectiveness. Furthermore, it could be possible that the effectiveness of interventions to prevent depression was underestimated, because the interventions to prevent depression could be less effective in depressed women [58].

Finally, this umbrella review examined the effectiveness of all types of preventive interventions versus control groups (e.g., usual care or placebo). Thus, it should not be used to draw inferences about the comparative effectiveness of the interventions included [19]. Future research should evaluate the effectiveness of specific preventive interventions for perinatal depression compared to others.

### 5.4 Comparison with existing literature

Our findings are consistent with the evidence from multiple overviews and meta-analyses that physical activity has a protective role in reducing the risk of depression in the general population [22,59,60]. We found a trend towards greater effectiveness when physical activity targeted at-risk women with a history of depression or elevated depression symptoms during the perinatal period [46]. However, women may be less likely to undertake exercise due to the symptoms of depression (e.g., less energy, fatigue), which can be an added barrier for women to exercise during pregnancy [61]. Regarding the type of the exercise activity, our study cannot recommend any intervention in favour of others, but most trials were aerobic in nature [46,51]. Therefore, our results may support the current clinical guidelines of physical activity for pregnant and postpartum women [62,63].

It is widely accepted that psychological, psychosocial and/or educational interventions are effective ways to prevent depression [58,64,65] and our results may support that this knowledge is also valid for perinatal depression. The extensive revision by the US Preventive Task Force reached the same conclusion, in particular depression focused cognitive behavioural and interpersonal therapies [66,67] for at-risk women. Within the psychological classification, the SR/MA included a wide variety of interventions (e.g., cognitive behavioural therapy, infant sleep, home support, educational programs), with no support for any type of therapy. In any case, other overviews have not found superiority of any psychological interventions over others in the case of therapy for perinatal depression [68]. We also found a trend that targeted programs are more effective than universal programs in the case of psychological interventions [47]. This can be explained by the scarcity of evidence on universal interventions during the perinatal period.

Concerning our results on pharmacological and dietary supplement interventions to prevent perinatal depression, our umbrella review found very limited evidence for the prevention of perinatal depression. These negative findings have also been found for the prevention of depression in the general population [65], so for now these interventions cannot be considered and more evidence is needed.

### 5.5 Practical implications

From the present umbrella review we can support exercise/physical activity based and psychological interventions for the reduction of symptoms of perinatal depression; however, due to the low methodological quality of the SR&MA included and the low quality of the evidence, the strength of this recommendation would initially be weak.

Current public health guidelines for physical exercise recommend 150 min of moderate activity or 75 min of vigorous activity per week (or some combination of these) for all pregnant and postpartum women [62,63]. Therefore, this recommendation would be easy to implement in health care as a universal strategy for the prevention of perinatal depression [18]. Given that the benefits of the exercise as regular activity have been shown, it is important that health professionals empower women to find a modality of exercise to which they will adhere over the long term [61].

Psychological preventive interventions for women with increased risk of depression (previous episodes or increased depressive symptoms in pregnancy) are recommended by the US Preventive Task Force [66,67] and by five clinical practice guidelines in Europe [18]. With the available evidence, we encourage this recommendation to be taken into account by future guide developers, as the evidence to implement psychological interventions at a universal level is still limited [47]. The implementation of population-based preventive interventions for perinatal depression in health care could bring large benefits to the community in terms of increased health, quality of life and cost reduction [15,69].

Finally, there is not enough evidence to formulate definite practical recommendations regarding pharmacological and dietary supplementary interventions. These results are in line with previous international recommendations [66,67,70].

### 5.6 Future research

There is need for high-quality SR&MA including powered RCTs assessing universal interventions and RCTs that excluded women with perinatal depression at baseline. Further research is required to address the existing barriers to implementing preventive interventions. For instance, harnessing the reach of digital technologies may present a new option for scaling up preventive interventions, which may be particularly useful for low- and middle-income settings. In addition, more economic evaluations should be conducted in tandem with RCTs in order to assess the cost effectiveness for the implementation of preventive interventions in maternity care.

## Ethics statement

Due to the characteristics of this study, an ethical assessment was not required.

## Consent statement

Due to the characteristics of this study, a consent form was not required.

## Funding

This publication is based upon work from COST Action Research Innovation and Sustainable Pan-European Network in Peripartum Depression Disorder (Riseup-PPD), CA18138, supported by COST (European Cooperation in Science and Technology).

## Author contribution

EM and SC-C designed the study. EM drafted the manuscript, and all the authors conducted a critical revision of the manuscript for important intellectual content. EM, PM-P and SC-C independently screened the potential database studies. RB, AK, H-NL, VM, PM-P, DO, and MFR extracted characteristics and assessed the methodological quality. EM and SC-C independently assessed overlap and statistical analysis. All the authors read, provided feedback, discussed, and approved the final manuscript.

## Declaration of Competing Interest

None.

## Data Availability

Data will be made available on request.

## Appendix A Supplementary data

Supplementary material 1 Image 1

Supplementary material 2 Image 2
