# Supplementary material for: Effectiveness of interventions to prevent perinatal depression: An umbrella review of systematic reviews and meta-analysis
Source: Gen Hosp Psychiatry. 2023 May-Jun;82:47–61. doi: 10.1016/j.genhosppsych.2023.03.007 (PMC10183436; doi:10.1016/j.genhosppsych.2023.03.007)
Supplement: Supplementary file 2 — Supplementary material 2 [file mmc2.docx]

**Table S1**

| Criteria | Inclusion criteria | Exclusion criteria |
| --- | --- | --- |
| Population | Perinatal women (from pregnancy to one year postpartum) | Perinatal women with depression |
| Intervention | All kinds of preventive interventions | None |
| Comparison | Care as usual, no treatment, waiting list, attention control or any type of placebo | Any type of intervention with available evidence of its  effectiveness in preventing depression. |
| Primary outcomes | Perinatal depression (postpartum depression and/or prenatal depression) through the incidence and/or reduction of symptomatology. | None |
| Type of design | Systematic review or/and  Meta-analysis of randomised controlled trials | None |
| Years of publication | No limit |  |
| Publication type | - Published reviews - Unpublished reviews | None |
| Language | No limit |  |

Eligible criteria for the umbrella review of SR/MA of preventive interventions for perinatal depression

**Table S2**

Search terms in PubMed, PsycINFO, Cochrane Database of Systematic Reviews and OpenGrey.

| **PubMed**: 01/12/2022 | |
| --- | --- |
| **Search terms** | **Items found** |
| #1.- Perinatal period:  ("postpartum period"[MeSH Terms]) OR (postpartum[Title/Abstract]) OR (postnatal[Title/Abstract]) OR (puerperal[Title/Abstract]) OR ("peripartum period"[MeSH Terms]) OR (peripartum[Title/Abstract]) OR (prenatal[Title/Abstract]) OR (antenatal[Title/Abstract]) OR ("pregnancy"[MeSH Terms]) OR ("pregnancy"[Title/Abstract]) OR (intrapartum[Title/Abstract]) OR ("pregnant women"[MeSH Terms]) OR ("pregnant women"[Title/Abstract]) | 1,238,696 |
| #2.- Depression:  (“depression, postpartum”[MeSH Terms]) OR (“depression, postpartum"[Title/Abstract]) OR ("depressive disorder"[MeSH Terms]) OR ("depressive disorder"[Title/Abstract]) OR (“depression” [MeSH Terms]) OR (“depression”[Title/Abstract]) OR (depress*[Title/Abstract]) | 587,588 |
| #3.- Prevention:  ((prevent*[Title/Abstract]) OR (incidence[Title/Abstract]) OR (symptom*[Title/Abstract]) OR (intervention*[Title/Abstract])) | 4,519,193 |
| #4.- Systematic review:  (((systematic review[ti] OR systematic literature review[ti] OR systematic scoping review[ti] OR systematic narrative review[ti] OR systematic qualitative review[ti] OR systematic evidence review[ti] OR systematic quantitative review[ti] OR systematic meta-review[ti] OR systematic critical review[ti] OR systematic mixed studies review[ti] OR systematic mapping review[ti] OR systematic cochrane review[ti] OR systematic search and review[ti] OR systematic integrative review[ti]) NOT comment[pt] NOT (protocol[ti] OR protocols[ti])) NOT MEDLINE [subset]) OR (Cochrane Database Syst Rev[ta] AND review[pt]) OR systematic review[pt] | 249,507 |
| #5.- Combination of #1 AND #2 AND #3 AND #4:  (("postpartum period"[MeSH Terms]) OR (postpartum[Title/Abstract]) OR (postnatal[Title/Abstract]) OR (puerperal[Title/Abstract]) OR ("peripartum period"[MeSH Terms]) OR (peripartum[Title/Abstract]) OR (prenatal[Title/Abstract]) OR (antenatal[Title/Abstract]) OR ("pregnancy"[MeSH Terms]) OR ("pregnancy"[Title/Abstract]) OR (intrapartum[Title/Abstract]) OR ("pregnant women"[MeSH Terms]) OR ("pregnant women"[Title/Abstract])) AND ((“depression, postpartum”[MeSH Terms]) OR (“depression, postpartum"[Title/Abstract]) OR ("depressive disorder"[MeSH Terms]) OR ("depressive disorder"[Title/Abstract]) OR (“depression” [MeSH Terms]) OR (“depression”[Title/Abstract]) OR (depress*[Title/Abstract])) AND ((prevent*[Title/Abstract]) OR (incidence[Title/Abstract]) OR (symptom*[Title/Abstract]) OR (intervention*[Title/Abstract])) AND ((((systematic review[ti] OR systematic literature review[ti] OR systematic scoping review[ti] OR systematic narrative review[ti] OR systematic qualitative review[ti] OR systematic evidence review[ti] OR systematic quantitative review[ti] OR systematic meta-review[ti] OR systematic critical review[ti] OR systematic mixed studies review[ti] OR systematic mapping review[ti] OR systematic cochrane review[ti] OR systematic search and review[ti] OR systematic integrative review[ti]) NOT comment[pt] NOT (protocol[ti] OR protocols[ti])) NOT MEDLINE [subset]) OR (Cochrane Database Syst Rev[ta] AND review[pt]) OR systematic review[pt]) | 701 |
| **PsycINFO:** 01/12/2022 | |
| **Search terms** | **Items found** |
| #1.- Perinatal period:  (if(postnatal period) OR ti,ab(postnatal) OR if(perinatal period) OR ti,ab(perinatal) OR ti,ab(peripartum) OR if(antepartum period) OR ti,ab(antepartum) OR if(pregnancy) OR ti,ab(pregnan*) OR ti,ab(antepartum) OR if(intrapartum period) OR ti,ab(intrapartum)) | 74,788 |
| #2.- Depression:  if(depression) OR ti,ab(depression) OR if(puerperal depression) OR ti,ab(puerperal depression) OR if(postnatal depression) OR ti,ab(postnatal depression) OR if(postpartum depression) OR ti,ab(postpartum depression) OR if(major depression) OR ti,ab(major depression) OR ti,ab(depress*) | 374,943 |
| #3.- Prevention:  ti,ab(prevent*) OR ti,ab(incidence) OR ti,ab(symptom*) OR ti,ab(intervention*) | 260 |
| #4.- Systematic review:  if(systematic review) OR ti,ab(systematic review) OR if(literature review) OR ti,ab(literature review) OR if(meta analysis) OR ti,ab(meta analysis) OR if(review) OR ti,ab(review) | 664,001 |
| #5.- Combination of #1 AND #2 AND #3 AND #4:  (if(postnatal period) OR ti,ab(postnatal) OR if(perinatal period) OR ti,ab(perinatal) OR ti,ab(peripartum) OR if(antepartum period) OR ti,ab(antepartum) OR if(pregnancy) OR ti,ab(pregnan*) OR ti,ab(antepartum) OR if(intrapartum period) OR ti,ab(intrapartum)) AND (if(depression) OR ti,ab(depression) OR if(puerperal depression) OR ti,ab(puerperal depression) OR if(postnatal depression) OR ti,ab(postnatal depression) OR if(postpartum depression) OR ti,ab(postpartum depression) OR if(major depression) OR ti,ab(major depression) OR ti,ab(depress*)) AND (ti,ab(prevent*) OR ti,ab(incidence) OR ti,ab(symptom*) OR ti,ab(intervention*)) AND (if(systematic review) OR ti,ab(systematic review) OR if(literature review) OR ti,ab(literature review) OR if(meta analysis) OR ti,ab(meta analysis) OR if(review) OR ti,ab(review)) | 478 |
| **Cochrane Database of Systematic Reviews:** 24/03/2022 | |
| **Search terms** | **Items found** |
| #1.- Perinatal period:  "postpartum period":ti,ab,kw or "postpatum":ti,ab or "postnatal":ti,ab or "puerperal":ti,ab or "peripartum period":ti,ab,kw or "peripartum":ti,ab or "prenatal":ti,ab or "antenatal":ti,ab or "pregnancy":ti,ab,kw or "intrapartum":ti,ab or "pregnant women":ti,ab,kw | 1189 |
| #2.- Depression:  "postpartum depression":ti,ab,kw or "depression":ti,ab,kw or "depressive disorder":ti,ab,kw or “major depressive disorder”:ti,ab,kw or depress*:ti,ab,kw | 750 |
| #3.- Prevention:  prevent*:ti,ab,kw or incidence:ti,ab or symptom*:ti,ab or intervention*:ti,ab | 7446 |
| #4.- Combination of #1 AND #2 AND #3:  ("postpartum period":ti,ab,kw or "postpatum":ti,ab or "postnatal":ti,ab or "puerperal":ti,ab or "peripartum period":ti,ab,kw or "peripartum":ti,ab or "prenatal":ti,ab or "antenatal":ti,ab or "pregnancy":ti,ab,kw or "intrapartum":ti,ab or "pregnant women":ti,ab,kw) AND ("postpartum depression":ti,ab,kw or "depression":ti,ab,kw or "depressive disorder":ti,ab,kw or “major depressive disorder”:ti,ab,kw or depress*:ti,ab,kw) AND (prevent*:ti,ab,kw or incidence:ti,ab or symptom*:ti,ab or intervention*:ti,ab) | 90 |
| **OpenGrey (via DANS EASY):** 01/12/2022 | |
| **Search terms** | **Items found** |
| (depress* OR postpartum depression OR perinatal depression OR postnatal depression) AND (postpartum OR postnatal OR perinatal OR peripartum OR pregnan*) AND (prevent* OR symptom* OR incidence) | 33 |

Table S3. Excluded systematic reviews and meta-analyses with reasons (N=107).

| **Excluded studies** | |
| --- | --- |
| **Study** | **Reasons for exclusion** |
| 1. Adina, J., Morawska, A., Mitchell, A. E., & McBryde, M. (2022). Effect of Parenting Interventions on Perinatal Depression and Implications for Infant Developmental Outcomes: A Systematic Review and Meta-Analysis. Clinical Child and Family Psychology Review, 25(2), 316–338. https://doi.org/10.1007/s10567-021-00371-3 | It is not included only RCTs. |
| 1. Alves, S., Martins, A., Fonseca, A., Canavarro, M. C., & Pereira, M. (2018). Preventing and Treating Women’s Postpartum Depression: A Qualitative Systematic Review on Partner-Inclusive Interventions. *Journal of Child and Family Studies,* 27, 1-25. https://doi.org/10.1007/s10826-017-0889-z | It is not included only RCTs. |
| 1. Ashford, M. T., Olander, E. K., & Ayers, S. (2016). Computer- or web-based interventions for perinatal mental health: A systematic review*. Journal of Affective Disorders, 197*, 134–146. https://doi.org/10.1016/j.jad.2016.02.057 | It is not included only RCTs. |
| 1. Austin, M. P. (2003). Targeted group antenatal prevention of postnatal depression: A review. *Acta Psychiatrica Scandinavica, 107(4),* 244–250. https://doi.org/10.1034/j.1600-0447.2003.00086.x | It is not included only RCTs. |
| 1. Austin, M. P., Priest, S. R., & Sullivan, E. A. (2008). Antenatal psychosocial assessment for reducing perinatal mental health morbidity. Cochrane Database of Systematic Reviews, (4). https://doi.org/10.1002/14651858.CD005124.pub2 | It only included one trial on the prevention of perinatal depression. |
| 1. Bastos, M. H., Furuta, M., Small, R., Mckenzie-Mcharg, K., & Bick, D. (2015). Debriefing interventions for the prevention of psychological trauma in women following childbirth. *Cochrane Database of Systematic Reviews*. https://doi.org/10.1002/14651858.CD007194.pub2 | It is not included only RCTs. |
| 1. Borja-Hart, N. L., & Marino, J. (2010). Role of omega-3 fatty acids for prevention or treatment of perinatal depression. *Pharmacotherapy, 30(2),* 210–216. https://doi.org/10.1592/phco.30.2.210 | It is not a systematic review or meta-analysis. |
| 1. Buultjens, M., Farouque, A., Karimi, L., Whitby, L., Milgrom, J., & Erbas, B. (2020, December 25). The contribution of group prenatal care to maternal psychological health outcomes: A systematic review. *Women and Birth*. https://doi.org/10.1016/j.wombi.2020.12.004 | It is not included only RCTs. |
| 1. Camacho, E. M., & Shields, G. E. (2018). Cost-effectiveness of interventions for perinatal anxiety and/or depression: A systematic review. *BMJ Open, 8(8),* 1–10. https://doi.org/10.1136/bmjopen-2018-022022 | It is not focused on the prevention of perinatal depression. |
| 1. Catling, C. J., Medley, N., Foureur, M., Ryan, C., Leap, N., Teate, A., & Homer, C. S. E. (2015). Group versus conventional antenatal care for women. *Cochrane Database of Systematic Reviews,* 2015(2). https://doi.org/10.1002/14651858.CD007622.pub3 | It only included one trial on the prevention of perinatal depression. |
| 1. Chae, J., & Kim, H. K. (2021). Internet-based prenatal interventions for maternal health among pregnant women: A systematic review and meta-analysis. Children and Youth Services Review, 127, 10. https://doi.org/http://dx.doi.org/10.1016/j.childyouth.2021.106079 | It is not focused on the prevention of perinatal depression. |
| 1. Chan, C. W. H., Yeung, E. A., & Law, B. M. H. (2019). Effectiveness of physical activity interventions on pregnancy-related outcomes among pregnant women: A systematic review. *International Journal of Environmental Research and Public Health, 16(10)*. https://doi.org/10.3390/ijerph16101840 | It is not focused on the prevention of perinatal depression. |
| 1. Clatworthy J. The effectiveness of antenatal interventions to prevent postnatal depression in high-risk women. J Affect Disord [Internet]. 2012 Mar;137(1–3):25–34. http://dx.doi.org/10.1016/j.jad.2011.02.029 | It is not a systematic review or meta-analysis. |
| 1. Ciappolino, V., Delvecchio, G., Agostoni, C., Mazzocchi, A., Altamura, A. C., & Brambilla, P. (2017). The role of n-3 polyunsaturated fatty acids (n-3PUFAs) in affective disorders. *Journal of Affective Disorders*, *224*, 32–47. https://doi.org/10.1016/j.jad.2016.12.034 | It is not focused on the prevention of perinatal depression. |
| 1. Claridge, A. M. (2014). Efficacy of systemically oriented psychotherapies in the treatment of perinatal depression: A meta-analysis. *Archives of Women’s Mental Health, 17(1),* 3–15. https://doi.org/10.1007/s00737-013-0391-6 | It is not focused on the prevention of perinatal depression. |
| 1. Corbally, L., & Wilkinson, M. (2021). The Effect of Mindfulness-Based Interventions on Stress, Depression and Anxiety During the Perinatal Period in Women Without Pre-existing Stress, Depressive or Anxiety Disorders: a Systematic Review and Meta-analysis of Controlled Trials. Mindfulness, 12(10), 2357–2370. https://doi.org/10.1007/s12671-021-01697-3 | It is not included only RCTs. |
| 1. Daley, A. J., Foster, L., Long, G., Palmer, C., Robinson, O., Walmsley, H., & Ward, R. (2015). The effectiveness of exercise for the prevention and treatment of antenatal depression: Systematic review with meta-analysis. *BJOG: An International Journal of Obstetrics and Gynaecology, 122(1),* 57–62. https://doi.org/10.1111/1471-0528.12909 | It only included one trial on the prevention of perinatal depression. |
| 1. Davenport, M. H., McCurdy, A. P., Mottola, M. F., Skow, R. J., Meah, V. L., Poitras, V. J., … Ruchat, S.-M. (2018). Impact of prenatal exercise on both prenatal and postnatal anxiety and depressive symptoms: a systematic review and meta-analysis. *British Journal of Sports Medicine, 52(21),* 1376–1385. https://doi.org/10.1136/bjsports-2018-099697 | It is not included only RCTs. |
| 1. Dennis, C.-L. E. (2004). Preventing Postpartum Depression Part I: A Review of Biological Interventions. *The Canadian Journal of Psychiatry, 49(7),* 467–475. https://doi.org/10.1177/070674370404900708 | It is not included only RCTs. |
| 1. Dennis, C. (2005). Postnatal Depression: Systematic Review. *British Medical Journal, 331(July)*, 1–8. | It is updated by Dennis et al. (2013). |
| 1. Dennis, C.-L., & Kingston, D. (2008). A Systematic Review of Telephone Support for Women During Pregnancy and the Early Postpartum Period. *Journal of Obstetric, Gynecologic & Neonatal Nursing*, *37*(3), 301–314. https://doi.org/10.1111/j.1552-6909.2008.00235.x | It is not focused on the prevention of perinatal depression. |
| 1. Dennis, C.-L., Ross, L. E., & Herxheimer, A. (2008). Oestrogens and progestins for preventing and treating postpartum depression. *Cochrane Database of Systematic Reviews, (4).* https://doi.org/10.1002/14651858.CD001690.pub2 | It only included one trial on the prevention of perinatal depression. |
| 1. Dhillon, A., Sparkes, E., & Duarte, R. V. (2017). Mindfulness-Based Interventions During Pregnancy: a Systematic Review and Meta-analysis. *Mindfulness, 8(6),* 1421–1437. https://doi.org/10.1007/s12671-017-0726-x | It is not focused on the prevention of perinatal depression. |
| 1. Dimidjian, S., & Goodman, S. H. (2009). Nonpharmacologic Intervention and Prevention Strategies for Depression During Pregnancy and the Postpartum*. Clinical Obstetrics and Gynecology, 52(3), 498–515. https://doi.org/10.1097/GRF.0b013e3181b52da6* | It is not a systematic review or meta-analysis. |
| 1. Dol, J., Richardson, B., Murphy, G. T., Aston, M., McMillan, D., & Campbell-Yeo, M. (2020). Impact of mobile health interventions during the perinatal period on maternal psychosocial outcomes: a systematic review. *JBI Database of Systematic Reviews and Implementation Reports, 18(1),* 30–55. https://doi.org/10.11124/JBISRIR-D-19-00191 | It is not included only RCTs. |
| 1. Douglas, P. S., & Hill, P. S. (2013). Behavioral sleep interventions in the first six months of life do not improve outcomes for mothers or infants: A systematic review. *Journal of Developmental and Behavioral Pediatrics, 34(7),* 497–507. https://doi.org/10.1097/DBP.0b013e31829cafa6 | It is not included only RCTs. |
| 1. Evans, K., Spiby, H., & Morrell, J. C. (2020). Non-pharmacological interventions to reduce the symptoms of mild to moderate anxiety in pregnant women. A systematic review and narrative synthesis of women’s views on the acceptability of and satisfaction with interventions. *Archives of Women’s Mental Health, 23(1),* 11–28. https://doi.org/10.1007/s00737-018-0936-9 | It is not focused on the prevention of perinatal depression. |
| 1. Evans, K., Rennick-Egglestone, S., Cox, S., Kuipers, Y., & Spiby, H. (2022). Remotely Delivered Interventions to Support Women With Symptoms of Anxiety in Pregnancy: Mixed Methods Systematic Review and Meta-analysis. Journal of Medical Internet Research, 24(2), e28093. https://doi.org/10.2196/28093 | It is not focused on the prevention of perinatal depression. |
| 1. Fassaie, S., & McAloon, J. (2020). Maternal distress, HPA activity, and antenatal interventions: A systematic review. *Psychoneuroendocrinology*, *112*(February), 104477. https://doi.org/10.1016/j.psyneuen.2019.104477 | It is not focused on the prevention of perinatal depression. |
| 1. Fontein-Kuipers, Y. J., Nieuwenhuijze, M. J., Ausems, M., Budé, L., & De Vries, R. (2014). Antenatal interventions to reduce maternal distress: A systematic review and meta-analysis of randomised trials. *BJOG: An International Journal of Obstetrics and Gynaecology*, *121*(4), 389–397. https://doi.org/10.1111/1471-0528.12500 | It is not focused on the prevention of perinatal depression. |
| 1. Gamble, J. A., Creedy, D. K., Webster, J., & Moyle, W. (2002). A review of the literature on debriefing or non-directive counselling to prevent postpartum emotional distress. *Midwifery, 18(1),* 72–79. https://doi.org/10.1054/midw.2001.0287 | It is not included only RCTs. |
| 1. Garcia, E. R., & Yim, I. S. (2017). A systematic review of concepts related to women’s empowerment in the perinatal period and their associations with perinatal depressive symptoms and premature birth. *BMC Pregnancy and Childbirth, 17(Suppl 2).* https://doi.org/10.1186/s12884-017-1495-1 | It is not focused on the prevention of perinatal depression. |
| 1. Gilmore, B., & McAuliffe, E. (2013). Effectiveness of community health workers delivering preventive interventions for maternal and child health in low- and middle-income countries: A systematic review. *BMC Public Health*, *13*(1), 1. https://doi.org/10.1186/1471-2458-13-847 | It only included one trial on the prevention of perinatal depression. |
| 1. Gould, J. F., Best, K., & Makrides, M. (2017). Perinatal nutrition interventions and post-partum depressive symptoms. *Journal of Affective Disorders*, *224*(July), 2–9. https://doi.org/10.1016/j.jad.2016.12.014 | It is not a systematic review or meta-analysis. |
| 1. Grigoriadis, S., Robinson, G. E., Fung, K., Ross, L. E., Chee, C., Yin I., Dennis, C., & Romans, S. (2009). Traditional Postpartum Practices and Rituals: Clinical Implications. *The Canadian Journal of Psychiatry*, *54*(12), 834–840. https://doi.org/10.1177/070674370905401206 | It is not focused on the prevention of perinatal depression. |
| 1. Gurung, B., Jackson, L. J., Monahan, M., Butterworth, R., & Roberts, T. E. (2018). Identifying and assessing the benefits of interventions for postnatal depression: A systematic review of economic evaluations. *BMC Pregnancy and Childbirth*, *18*(1). https://doi.org/10.1186/s12884-018-1738-9 | It is not focused on the prevention of perinatal depression. |
| 1. Hall, H. G., Cant, R., Munk, N., Carr, B., Tremayne, A., Weller, C., … Lauche, R. (2020). The effectiveness of massage for reducing pregnant women’s anxiety and depression; systematic review and meta-analysis. *Midwifery, 90,* 102818. https://doi.org/10.1016/j.midw.2020.102818 | It is not included only RCTs. |
| 1. Hanach, N., de Vries, N., Radwan, H., & Bissani, N. (2021). The effectiveness of telemedicine interventions, delivered exclusively during the postnatal period, on postpartum depression in mothers without history or existing mental disorders: A systematic review and meta-analysis. Midwifery, 94, 102906. https://doi.org/10.1016/j.midw.2020.102906 | It is not focused on the prevention of perinatal depression. |
| 1. Hsu, M. C., Tung, C. Y., & Chen, H. E. (2018). Omega-3 polyunsaturated fatty acid supplementation in prevention and treatment of maternal depression: Putative mechanism and recommendation. *Journal of Affective Disorders, 238,* 47–61. https://doi.org/10.1016/j.jad.2018.05.018 | It is not included only RCTs. |
| 1. Huang, R., Yan, C., Tian, Y., Lei, B., Yang, D., Liu, D., & Lei, J. (2020). Effectiveness of peer support intervention on perinatal depression: A systematic review and meta-analysis*. Journal of Affective Disorders, 276,* 788–796. https://doi.org/10.1016/j.jad.2020.06.048 | The results of prevention of perinatal depression were not provided separately. |
| 1. Hussain, T., Smith, P., & Yee, L. M. (2020). Mobile Phone–Based Behavioral Interventions in Pregnancy to Promote Maternal and Fetal Health in High-Income Countries: Systematic Review. *JMIR MHealth and UHealth,* 8(5), e15111. https://doi.org/10.2196/15111 | It is not focused on the prevention of perinatal depression. |
| 1. Iwata, H., Mori, E., Maehara, K., Harada, N., & Saito, A. (2021). Effectiveness of parenting education for expectant primiparous women in Asia: a systematic review. JBI Evidence Synthesis, 19(3), 523–555. https://doi.org/10.11124/JBISRIR-D-19-00327 | It is not included only RCTs. |
| 1. Jidong, D. E., Husain, N., Roche, A., Lourie, G., Ike, T. J., Murshed, M., Park, M. S., Karick, H., Dagona, Z. K., Pwajok, J. Y., Gumber, A., Francis, C., Nyam, P. P., & Mwankon, S. B. (2021). Psychological interventions for maternal depression among women of African and Caribbean origin: a systematic review. BMC Women’s Health, 21(1), 1–14. https://doi.org/10.1186/s12905-021-01202-x | It is not focused on the prevention of perinatal depression. |
| 1. Kawanishi, Y., Hanley, S. J. B., Tabata, K., Nakagi, Y., Ito, T., Yoshioka, E., … Saijo, Y. (2015). Effects of prenatal yoga: a systematic review of randomized controlled trials. *[Nihon Kōshū Eisei Zasshi] Japanese Journal of Public Health, Vol. 62, pp. 221–231.* https://doi.org/10.11236/jph.62.5_221 | It is not focused on the prevention of perinatal depression. |
| 1. Kraljevic, M., & Warnock, F. F. (2013). Early educational and behavioral RCT interventions to reduce maternal symptoms of psychological trauma following preterm birth: A systematic review. *Journal of Perinatal and Neonatal Nursing, 27(4),* 311–327. https://doi.org/10.1097/JPN.0b013e3182a8bfe2 | It is not focused on the prevention of perinatal depression. |
| 1. Kwon, R., Kasper, K., London, S., & Haas, D. M. (2020). A systematic review: The effects of yoga on pregnancy. *European Journal of Obstetrics, Gynecology, and Reproductive Biology, 250,* 171–177. https://doi.org/10.1016/j.ejogrb.2020.03.044 | It is not focused on the prevention of perinatal depression. |
| 1. Lara-Cinisomo, S., Ramirez Olarte, A., Rosales, M., & Barrera, A. Z. (2021). A Systematic Review of Technology-Based Prevention and Treatment Interventions for Perinatal Depression and Anxiety in Latina and African American Women. In Maternal and Child Health Journal. Springer. https://doi.org/10.1007/s10995-020-03028-9 | It is not included only RCTs. |
| 1. Lavender, T., Richens, Y., Milan, S. J., Smyth, R. M., & Dowswell, T. (2013). Telephone support for women during pregnancy and the first six weeks postpartum. *Cochrane Database of Systematic Reviews, 2013(7).* https://doi.org/10.1002/14651858.CD009338.pub2 | It is not focused on the prevention of perinatal depression. |
| 1. Lau, Y., Htun, T. P., Wong, S. N., Tam, W. S. W., & Klainin-Yobas, P. (2017). Therapist-supported internet-based cognitive behavior therapy for stress, anxiety, and depressive symptoms among postpartum women: A systematic review and meta-analysis. *Journal of Medical Internet Research, 19(4)*, 1–18. https://doi.org/10.2196/jmir.6712 | It is not focused on the prevention of perinatal depression. |
| 1. Lau, Y., Cheng, J.-Y., Wong, S.-H., Yen, K.-Y., & Cheng, L.-J. (2021). Effectiveness of digital psychotherapeutic intervention among perinatal women: A systematic review and meta-analysis of randomized controlled trials. World Journal of Psychiatry, 11(4), 133–152. https://doi.org/10.5498/wjp.v11.i4.133 | It is not focused on the prevention of perinatal depression. |
| 1. Lau, Y., Yen, K. Y., Wong, S. H., Cheng, J. Y., & Cheng, L. J. (2022). Effect of digital cognitive behavioral therapy on psychological symptoms among perinatal women in high income-countries: A systematic review and meta-regression. Journal of Psychiatric Research, 146, 234–248. https://doi.org/10.1016/j.jpsychires.2021.11.012 | It is not focused on the prevention of perinatal depression. |
| 1. Laurenzi, C. A., Gordon, S., Abrahams, N., du Toit, S., Bradshaw, M., Brand, A., Melendez-Torres, G. J., Tomlinson, M., Ross, D. A., Servili, C., Carvajal-Aguirre, L., Lai, J., Dua, T., Fleischmann, A., & Skeen, S. (2020). Psychosocial interventions targeting mental health in pregnant adolescents and adolescent parents: a systematic review. Reproductive Health, 17(1), 65. https://doi.org/10.1186/s12978-020-00913-y | The results of prevention of perinatal depression were not provided separately. |
| 1. Lee, E. W., Denison, F. C., Hor, K., & Reynolds, R. M. (2016). Web-based interventions for prevention and treatment of perinatal mood disorders: A systematic review. *BMC Pregnancy and Childbirth*, *16*(1). https://doi.org/10.1186/s12884-016-0831-1 | It only included one trial on the prevention of perinatal depression. |
| 1. Leis, J. A., Mendelson, T., Tandon, S. D., & Perry, D. F. (2009). A systematic review of home-based interventions to prevent and treat postpartum depression. *Archives of Women’s Mental Health, 12*(1), 3–13. https://doi.org/10.1007/s00737-008-0039-0 | It only included one trial on the prevention of perinatal depression. |
| 1. Li, Z., Liu, Y., Wang, J., Liu, J., Zhang, C., & Liu, Y. (2020). Effectiveness of cognitive behavioural therapy for perinatal depression: A systematic review and meta‐analysis. Journal of Clinical Nursing, 29(17–18), 3170–3182. https://doi.org/10.1111/jocn.15378 | It is not focused on the prevention of perinatal depression. |
| 1. Lieberman, K., Le, H. N., & Perry, D. F. (2014). A systematic review of perinatal depression interventions for adolescent mothers. *Journal of Adolescence*. https://doi.org/10.1016/j.adolescence.2014.08.004 | It is not included only RCTs. |
| 1. Mahdi, A., Dembinsky, M., Bristow, K., & Slade, P. (2019). Approaches to the prevention of postnatal depression and anxiety – a review of the literature. *Journal of Psychosomatic Obstetrics & Gynecology, 40*(4), 250–263. https://doi.org/10.1080/0167482X.2018.1512577 | It is not a systematic review or meta-analysis. |
| 1. Marc, I., Blanchet, C., Ernst, E., Hodnett, E. D., Turcot, L., & Dodin, S. (2009). Mind-body interventions during pregnancy for preventing or treating women’s anxiety. *Cochrane Database of Systematic Reviews, (1).* https://doi.org/10.1002/14651858.CD007559 | It only included one trial on the prevention of perinatal depression. |
| 1. Middleton, P., Jc, G., Jf, G., Shepherd, E., Sf, O., & Makrides, M. (2018). Omega-3 fatty acid addition during pregnancy (Review) summary of findings for the main comparison. *Cochrane Database Syst Rev.*, *15*(11), CD003402. https://doi.org/10.1002/14651858.CD003402.pub3.www.cochranelibrary.com | It is not focused on the prevention of perinatal depression. |
| 1. Miniati, M., Callari, A., Calugi, S., Rucci, P., Savino, M., Mauri, M., & Dell’Osso, L. (2014). Interpersonal psychotherapy for postpartum depression: A systematic review. *Archives of Women’s Mental Health, 17(4),* 257–268. https://doi.org/10.1007/s00737-014-0442-7 | It is not focused on the prevention of perinatal depression. |
| 1. Morrell, C. J., Sutcliffe, P., Booth, A., Stevens, J., Scope, A., Stevenson, M., … Stewart-Brown, S. (2016). A systematic review, evidence synthesis and meta-analysis of quantitative and qualitative studies evaluating the clinical effectiveness, the cost-effectiveness, safety and acceptability of interventions to prevent postnatal depression. *Health Technology Assessment*. https://doi.org/10.3310/hta20370 | It is not included only RCTs. |
| 1. Morres, I. D., Tzouma, N.-A., Hatzigeorgiadis, A., Krommidas, C., Kotronis, K. V, Dafopoulos, K., Theodorakis, Y., & Comoutos, N. (2022). Exercise for perinatal depressive symptoms: A systematic review and meta-analysis of randomized controlled trials in perinatal health services. *Journal of Affective Disorders,* 298(Part A), 26–42. https://doi.org/10.1016/j.jad.2021.10.124 | It is not focused on the prevention of perinatal depression. |
| 1. Mu, T.-Y., Li, Y.-H., Xu, R.-X., Chen, J., Wang, Y.-Y., & Shen, C.-Z. (2021). Internet-based interventions for postpartum depression: A systematic review and meta-analysis. *Nursing Open, 8*, 1125–1134. | It is not focused on the prevention of perinatal depression. |
| 1. Nakamura, A., van der Waerden, J., Melchior, M., Bolze, C., El-Khoury, F., & Pryor, L. (2019). Physical activity during pregnancy and postpartum depression: Systematic review and meta-analysis. *Journal of Affective Disorders*, *246*, 29–41. https://doi.org/10.1016/j.jad.2018.12.009 | It is not focused on the prevention of perinatal depression. |
| 1. Nardi B, Laurenzi S, Di Nicolò M, Bellantuono C. [Is the cognitive-behavioural therapy an effective strategy also in the prevention of postpartum depression? A critical review]. Riv Psichiatr [Internet]. 2012;47(3):205–13. http://www.ncbi.nlm.nih.gov/pubmed/22825435 | It is not a systematic review or meta-analysis. |
| 1. Newberry, S. J., Chung, M., Booth, M., Maglione, M. A., Tang, A. M., O'Hanlon, C. E., Wang, D. D., Okunogbe, A., Huang, C., Motala, A., Trimmer, M., Dudley, W., Shanman, R., Coker, T. R., & Shekelle, P. G. (2016). Omega-3 Fatty Acids and Maternal and Child Health: An Updated Systematic Review. *Evidence report/technology assessment, (224),* 1–826. https://doi.org/10.23970/AHRQEPCERTA224 | It is not focused on the prevention of perinatal depression. |
| 1. O’Connor, E., Senger, C. A., Henninger, M. L., Coppola, E., & Gaynes, B. N. (2019). Interventions to Prevent Perinatal Depression: Evidence Report and Systematic Review for the US Preventive Services Task Force. *JAMA - Journal of the American Medical Association, 321*(6), 588–601. https://doi.org/10.1001/jama.2018.20865 | It is not included only RCTs. |
| 1. Park, S., Kim, J., Oh, J., & Ahn, S. (2020). Effects of psychoeducation on the mental health and relationships of pregnant couples: A systemic review and meta-analysis*. International Journal of Nursing Studies, 104,* 103439. https://doi.org/10.1016/j.ijnurstu.2019.103439 | It is not focused on the prevention of perinatal depression. |
| 1. Pentland, V., Spilsbury, S., Biswas, A., Mottola, M. F., Paplinskie, S., & Mitchell, M. S. (2022). Does Walking Reduce Postpartum Depressive Symptoms? A Systematic Review and Meta-Analysis of Randomized Controlled Trials. *Journal of Women’s Health, 31(4),* 555–563. https://doi.org/10.1089/jwh.2021.0296 | It is not focused on the prevention of perinatal depression. |
| 1. Perry, M., Becerra, F., Kavanagh, J., Serre, A., Vargas, E., & Becerril, V. (2015). Community-based interventions for improving maternal health and for reducing maternal health inequalities in high-income countries: A systematic map of research. *Globalization and Health*. https://doi.org/10.1186/s12992-014-0063-y | It is not a systematic review or meta-analysis. |
| 1. Pezley, L. (2022). Online intervention to prevent perinatal depression and promote breastfeeding [the University of Illinois at Chicago]. In Dissertation Abstracts International: Section B: The Sciences and Engineering. https://www.proquest.com/dissertations-theses/online-intervention-prevent-perinatal-depression/docview/2596640262/se-2?accountid=14568 | It is not focused on the prevention of perinatal depression. |
| 1. Ponting, C., Mahrer, N. E., Zelcer, H., Dunkel Schetter, C., & Chavira, D. A. (2020). Psychological interventions for depression and anxiety in pregnant Latina and Black women in the United States: A systematic review. *Clinical Psychology and Psychotherapy, 27*(2), 249–265. https://doi.org/10.1002/cpp.2424 | It is not included only RCTs. |
| 1. Poyatos-León, R., García-Hermoso, A., Sanabria-Martínez, G., Álvarez-Bueno, C., Cavero-Redondo, I., & Martínez-Vizcaíno, V. (2017). Effects of exercise-based interventions on postpartum depression: A meta-analysis of randomized controlled trials. *Birth, 44*(3), 200–208. https://doi.org/10.1111/birt.12294 | It is not included only RCTs. |
| 1. Pritchett, R. V., Daley, A. J., & Jolly, K. (2017). Does aerobic exercise reduce postpartum depressive symptoms?: A systematic review and meta-analysis. *British Journal of General Practice, 67*(663), e684–e691. https://doi.org/10.3399/bjgp17X692525 | It is not focused on the prevention of perinatal depression. |
| 1. Rayce, S. B., Rasmussen, I. S., Væver, M. S., & Pontoppidan, M. (2020). Effects of parenting interventions for mothers with depressive symptoms and an infant: systematic review and meta-analysis. *BJPsych Open, 6*(1), e9. https://doi.org/10.1192/bjo.2019.89 | It is not focused on the prevention of perinatal depression. |
| 1. Rezaie-Keikhaie, K., Hastings-Tolsma, M., Bouya, S., Shad, F. S., Sari, M., Shoorvazi, M., … Balouchi, A. (2019). Effect of aromatherapy on post-partum complications: A systematic review. *Complementary Therapies in Clinical Practice, 35*(March), 290–295. https://doi.org/10.1016/j.ctcp.2019.03.010 | It is not focused on the prevention of perinatal depression. |
| 1. Rinaudo, L., & Hopwood, M. (2015). A narrative review of the efficacy of DHA for treatment of major depressive disorder and treatment and prevention of postnatal depression. *Advances in Integrative Medicine, 2*(1), 24–30. https://doi.org/10.1016/j.aimed.2015.02.004 | It is not a systematic review or meta-analysis. |
| 1. Roman, M., Constantin, T., & Bostan, C. M. (2020). The efficiency of online cognitive-behavioural therapy for postpartum depressive symptomatology: a systematic review and meta-analysis. *Women and Health*, *60*(1), 99–112. https://doi.org/10.1080/03630242.2019.1610824 | It is not focused on the prevention of perinatal depression. |
| 1. Saad, A., Magwood, O., Aubry, T., Alkhateeb, Q., Hashmi, S. S., Hakim, J., Ford, L., Kassam, A., Tugwell, P., & Pottie, K. (2021). Mobile interventions targeting common mental disorders among pregnant and postpartum women: An equity-focused systematic review. *PLOS ONE, 16*(10), e0259474. https://doi.org/10.1371/journal.pone.0259474 | It is not included only RCTs. |
| 1. Saccone, G., Saccone, I., & Berghella, V. (2016). Omega-3 long-chain polyunsaturated fatty acids and fish oil supplementation during pregnancy: Which evidence? *Journal of Maternal-Fetal and Neonatal Medicine, 29*(15), 2389–2397. https://doi.org/10.3109/14767058.2015.1086742 | It only included one trial on the prevention of perinatal depression. |
| 1. Sado, M., Ota, E., Stickley, A., & Mori, R. (2012). Hypnosis during pregnancy, childbirth, and the postnatal period for preventing postnatal depression. *Cochrane Database of Systematic Reviews*, (6). https://doi.org/10.1002/14651858.cd009062.pub2 | It only included one trial on the prevention of perinatal depression. |
| 1. Saligheh, M., Hackett, D., Boyce, P., & Cobley, S. (2017). Can exercise or physical activity help improve postnatal depression and weight loss? A systematic review. *Archives of Women’s Mental Health*, *20*(5), 595–611. https://doi.org/10.1007/s00737-017-0750-9 | It is not focused on the prevention of perinatal depression. |
| 1. Sánchez-Polán, M., Franco, E., Silva-José, C., Gil-Ares, J., Pérez-Tejero, J., Barakat, R., & Refoyo, I. (2021). Exercise During Pregnancy and Prenatal Depression: A Systematic Review and Meta-Analysis. *Frontiers in Physiology, 12,* 640024. https://doi.org/10.3389/fphys.2021.640024 | It is not focused on the prevention of perinatal depression. |
| 1. Sakamoto, J. L., Carandang, R. R., Kharel, M., Shibanuma, A., Yarotskaya, E., Basargina, M., & Jimba, M. (2022). Effects of mHealth on the psychosocial health of pregnant women and mothers: a systematic review. *BMJ Open, 12*, e056807–e056807. | It is not included only RCTs. |
| 1. Scope, A., Booth, A., Morrell, C. J., Sutcliffe, P., & Cantrell, A. (2017). Perceptions and experiences of interventions to prevent postnatal depression. A systematic review and qualitative evidence synthesis. *Journal of Affective Disorders.* https://doi.org/10.1016/j.jad.2016.12.017 It is not included only RCTs | It is not included only RCTs. |
| 1. Silang, K. A., Sohal, P. R., Bright, K. S., Leason, J., Roos, L., Lebel, C., Giesbrecht, G. F., & Tomfohr-Madsen, L. M. (2022). eHealth Interventions for Treatment and Prevention of Depression, Anxiety, and Insomnia During Pregnancy: Systematic Review and Meta-analysis. *JMIR Mental Health, 9*(2), e31116. https://doi.org/10.2196/31116 | The results of prevention of perinatal depression were not provided separately. |
| 1. Sockol, L. E. (2015). A systematic review of the efficacy of cognitive behavioral therapy for treating and preventing perinatal depression. *Journal of Affective Disorders*. https://doi.org/10.1016/j.jad.2015.01.052 | It is not included only RCTs. |
| 1. Sockol, L. E. (2018). A systematic review and meta-analysis of interpersonal psychotherapy for perinatal women. *Journal of Affective Disorders*, 232, 316–328. https://doi.org/10.1016/j.jad.2018.01.018 | It is not included only RCTs. |
| 1. Sockol, L. E., Neill Epperson, C., & Barber, J. P. (2013). Preventing postpartum depression: A meta-analytic review. *Clin Psychol Rev,* 33(8), 1205–1217. https://doi.org/10.1016/j.cpr.2013.10.004 | It is not included only RCTs. |
| 1. Shivakumar, G., Brandon, A. R., Snell, P. G., Santiago-Muñoz, P., Johnson, N. L., Trivedi, M. H., & Freeman, M. P. (2011). Antenatal depression: a rationale for studying exercise. *Depression and Anxiety*, *28*(3), 234–242. https://doi.org/10.1002/da.20777 | It is not focused on the prevention of perinatal depression. |
| 1. Shi, Z., & MacBeth, A. (2017). The Effectiveness of Mindfulness-Based Interventions on Maternal Perinatal Mental Health Outcomes: a Systematic Review. *Mindfulness*, *8*(4), 823–847. https://doi.org/10.1007/s12671-016-0673-y | It is not focused on the prevention of perinatal depression. |
| 1. Shimada, B. M. O., Santos, M. da S. O. M. Dos, Cabral, M. A., Silva, V. O., & Vagetti, G. C. (2021). Interventions among Pregnant Women in the Field of Music Therapy: A Systematic Review. *Revista Brasileira de Ginecologia e Obstetricia: Revista Da Federacao Brasileira Das Sociedades de Ginecologia e Obstetricia, 43*, 403–413. | It is not focused on the prevention of perinatal depression. |
| 1. Smith, K. S., Greene, M. W., Ramesh Babu, J., & Frugé, A. D. (2019). Psychobiotics as treatment for anxiety, depression, and related symptoms: a systematic review. *Nutritional Neuroscience*, *0*(0), 1–15. https://doi.org/10.1080/1028415X.2019.1701220 | It is not focused on the prevention of perinatal depression. |
| 1. Sparling, T. M., Henschke, N., Nesbitt, R. C., & Gabrysch, S. (2017). The role of diet and nutritional supplementation in perinatal depression: a systematic review. *Maternal and Child Nutrition, 13*(1), 1–36. https://doi.org/10.1111/mcn.12235 | It is not included only RCTs. |
| 1. Suto, M., Takehara, K., Yamane, Y., & Ota, E. (2017). Effects of prenatal childbirth education for partners of pregnant women on paternal postnatal mental health and couple relationship: A systematic review. *Journal of Affective Disorders, 210,* 115–121. https://doi.org/10.1016/j.jad.2016.12.025 | It is not focused on the prevention of perinatal depression. |
| 1. Tsai, S. S., Wang, H. H., & Chou, F. H. (2020). The effects of aromatherapy on postpartum women: A systematic review. *Journal of Nursing Research, 28*. https://doi.org/10.1097/jnr.0000000000000331 | It is not focused on the prevention of perinatal depression. |
| 1. Taylor, B. L., Cavanagh, K., & Strauss, C. (2016). The effectiveness of mindfulness-based interventions in the perinatal period: A systematic review and meta-analysis. *PLoS ONE, 11*(5), 1–30. https://doi.org/10.1371/journal.pone.0155720 | It is not included only RCTs. |
| 1. Teychenne, M., & York, R. (2013). Physical activity, sedentary behavior, and postnatal depressive symptoms: A review. *American Journal of Preventive Medicine, 45*(2), 217–227. https://doi.org/10.1016/j.amepre.2013.04.004 | It is not included only RCTs. |
| 1. Tong, P., Dong, L. P., Yang, Y., Shi, Y. H., Sun, T., & Bo, P. (2019). Traditional Chinese acupuncture and postpartum depression: A systematic review and meta-analysis. *Journal of the Chinese Medical Association, 82*(9), 719–726. https://doi.org/10.1097/JCMA.0000000000000140 | It is not focused on the prevention of perinatal depression. |
| 1. Van Kampen, M., Devoogdt, N., De Groef, A., Gielen, A., & Geraerts, I. (2015). The efficacy of physiotherapy for the prevention and treatment of prenatal symptoms: a systematic review. *International Urogynecology Journal, 26*(11), 1575–1586. https://doi.org/10.1007/s00192-015-2684-y | It only included one trial on the prevention of perinatal depression. |
| 1. Wadephul, F., Jones, C., & Jomeen, J. (2016). The Impact of Antenatal Psychological Group Interventions on Psychological Well-Being: A Systematic Review of the Qualitative and Quantitative Evidence. *Healthcare, 4*(2), 32. https://doi.org/10.3390/healthcare4020032 | It is not included only RCTs. |
| 1. Werner, E., Miller, M., Osborne, L. M., Kuzava, S., & Monk, C. (2015). Preventing postpartum depression: review and recommendations. Archives of *Women’s Mental Health, 18*(1), 41–60. https://doi.org/10.1007/s00737-014-0475-y | It is not a systematic review or meta-analysis. |
| 1. Wojcicki, J. M., & Heyman, M. B. (2011). Maternal omega-3 fatty acid supplementation and risk for perinatal maternal depression*. Journal of Maternal-Fetal and Neonatal Medicine, 24*(5), 680–686. https://doi.org/10.3109/14767058.2010.521873 | It is not included only RCTs. |
| 1. Wu, Q., Liu, Z., Pang, X., & Cheng, L. (2020). Efficacy of five-element music interventions in perinatal mental health and labor pain: A meta-analysis. *Complementary Therapies in Clinical Practice, 40,* 101217. https://doi.org/10.1016/j.ctcp.2020.101217 | It is not focused on the prevention of perinatal depression. |
| 1. Yang, L., Di, Y. M., Shergis, J. L., Li, Y., Zhang, A. L., Lu, C., … Xue, C. C. (2018). A systematic review of acupuncture and Chinese herbal medicine for postpartum depression. *Complementary Therapies in Clinical Practice*, *33*, 85–92. https://doi.org/10.1016/j.ctcp.2018.08.006 | It is not focused on the prevention of perinatal depression. |
| 1. Yonemoto, N., Dowswell, T., Nagai, S., & Mori, R. (2017). Schedules for home visits in the early postpartum period. *Cochrane Database of Systematic Reviews, 2017*(8). https://doi.org/10.1002/14651858.CD009326.pub3 | It is not focused on the prevention of perinatal depression. |
| 1. Yuan, M., Chen, H., Chen, D., Wan, D., Luo, F., Zhang, C., Nan, Y., Bi, X., & Liang, J. (2022). Effect of physical activity on prevention of postpartum depression: A dose-response meta-analysis of 186,412 women. *Frontiers in psychiatry, 13,* 984677. https://doi.org/10.3389/fpsyt.2022.984677 | It is not included only RCTs. |

Table S4. List of the countries of the unique RCTs (N=152) included in the umbrella review.

| Country | N |
| --- | --- |
| Australia | 24 |
| Brazil | 1 |
| Canada | 3 |
| China | 23 |
| Colombia | 1 |
| Denmark | 1 |
| English speaking countries | 1 |
| France | 1 |
| Hungary | 1 |
| India | 3 |
| Iran | 7 |
| Ireland | 1 |
| Japan | 2 |
| Kenya | 1 |
| Netherlands | 1 |
| New Zealand | 1 |
| Norway | 2 |
| Portugal | 1 |
| Romania | 1 |
| Saudi Arabia | 1 |
| Singapore | 1 |
| South Africa | 2 |
| Spain | 2 |
| Taiwan | 1 |
| United Kingdom | 18 |
| United States of America | 51 |
| Total | 152 |
